# Superior Energy Release of Ammonium Perchlorate Composites by Embedding Heterostructured Carbon Nanotube/Tricobalt Tetraoxide Thermal Conduction Pathways

**DOI:** 10.34133/research.0938

**Published:** 2025-10-15

**Authors:** Ruixuan Xu, Yuan Qin, Junlian Hao, Yongqiang Guo, Hao Jiang, Kejuan Meng, Sulan Yang, Kaili Zhang, Junwei Gu

**Affiliations:** ^1^ School of Chemistry and Chemical Engineering, Northwestern Polytechnical University, Xi’an, Shaanxi 710072, P. R. China.; ^2^ Department of Mechanical Engineering, City University of Hong Kong, Hong Kong 999077, P. R. China.; ^3^ National Key Laboratory of Solid Rocket Propulsion, Northwestern Polytechnical University, Xi’an, Shaanxi 710072, P. R. China.; ^4^ Aerospace College, University of Electronic Science and Technology of China, Chengdu, Sichuan 611731, P. R. China.

## Abstract

To address the inherent challenge of low reaction efficiency arising from interfacial thermal resistance in composite energetic materials, this study proposes a synergistic strategy integrating engineered thermal conduction pathways with precision catalysis for achieving high-efficiency energy release in ammonium perchlorate (AP). Heterostructured carbon nanotube (CNT)/Co_3_O_4_ was synthesized via in situ growth of Co_3_O_4_ nanoclusters on CNTs, followed by embedding onto the AP surface through spray drying-suspension coating technology. Comprehensive characterization confirmed the effective anchoring, uniform distribution, and interfacial interactions of Co_3_O_4_. With only 1 wt% CNT/Co_3_O_4_ loading, the high-temperature decomposition peak temperature of AP was dramatically reduced from 450.9 °C (pristine AP) to 310.7 °C, accompanied by a 24.3% enhancement in heat release and a substantial 64.8% reduction in activation energy. Combustion tests revealed a 72.6% increase in flame radiation intensity and a 2.3-fold acceleration in pressurization rate for AP@CNT/Co mixing with aluminum. Mechanistic studies elucidate a tripartite synergy: (a) CNT-derived thermal conduction pathways elevate thermal conductivity, (b) Co_3_O_4_ facilitates proton/electron transfer and drives the oxidation of gaseous products toward higher-valent nitrogen oxides, and (c) surface microporosity accelerates heat/mass diffusion. This concerted action enables focused, rapid, and efficient energy release from AP. This work establishes a generic interfacial engineering paradigm for enhancing energy release efficiency in composite energetic materials.

## Introduction

The advancement of aerospace propulsion technologies imposes stringent demands on solid propulsion systems, necessitating higher thrust-to-weight ratios and reduced structural loads. These requirements, in turn, elevate the performance standards for the energy release efficiency of solid propellants. As a representative high-solid-loading composite, solid propellants inherently contain multiple heterogeneous interfaces. The heat transfer inefficiency arising from interfacial thermal resistance markedly constrains reaction kinetics and energy release [1,2]. Ammonium perchlorate (AP), owing to its superior comprehensive properties, has become the predominant oxidizer, constituting over 50 wt% of propellant formulations. Consequently, heat transfer across the AP–binder and AP–fuel interfaces becomes a pivotal factor governing overall propellant performance. Developing advanced strategies to enhance the energy release efficiency of AP is therefore a crucial research objective for next-generation propulsion systems.

Substantial efforts have been dedicated to mitigating interfacial thermal resistance in composites, including incorporating highly thermally conductive fillers [3–5] and employing interfacial engineering strategies [6–8]. Carbon materials renowned for their high thermal conductivity (*λ*), such as graphene nanosheets (GNPs) and carbon nanotubes (CNTs). By constructing a 3-dimensional (3D) thermally conductive network with only 1 wt% GNPs, for example, Chen et al. [9] achieved a remarkable enhancement of over 70% in the *λ* of polymer-bonded explosives compared to the baseline counterpart. He et al. [10] assembled a hybrid 3D CNT/GNP network within polymer-bonded explosives, realizing a remarkable 176.5% enhancement in *λ* at a low loading of 1.31 vol%. Similarly, Liu et al. [11] and Shi et al. [12] further demonstrated that 2-dimensional (2D) carbon materials could effectively enhance the *λ* of solid propellants, leading to reduced ignition delay and higher burning rates.

Parallel to thermal management, catalytic decomposition of AP presents another avenue for enhancing energy release. AP undergoes 2 distinct decomposition stages upon heating: low-temperature decomposition (LTD) and high-temperature decomposition (HTD). A wide range of catalysts, including metal oxides [13–15], metal complexes [16,17], metal–organic frameworks [18–20], covalent organic frameworks [21], hydrogen-bonded organic frameworks [22], and carbon nanomaterial-based catalysts [23–27], have been explored to lower the HTD peak temperature and increase heat release. However, evaluations in most studies rely on conventional mechanical mixing, which often requires high catalyst loadings (up to 20 wt%) to achieve the desired effect. Such high loadings inevitably compromise the energy density and mechanical properties of propellants, which is a major drawback for practical applications.

It has been proved that the effect of catalysts can be largely influenced by dispersity and mixing uniformity [28]. Therefore, addressing this limitation, the emerging “precision catalysis” strategy has been proposed [29,30]. This approach emphasizes the precise spatial localization and controlled distribution of catalytic species near the reaction sites, enabling highly efficient catalysis at low loadings. For instance, confining graphene-based catalysts within aluminum (Al)@AP composites via spray-drying technology drastically reduced thermal decomposition activation energy by 67.4%, decreased ignition delay by 40.2%, and enhanced flame radiation intensity by 6.6-fold with merely 1 wt% catalyst [31]. This strategy effectively overcomes the drawbacks of traditional mixing by ensuring superior dispersion and intimate contact.

**Fig. 1.**
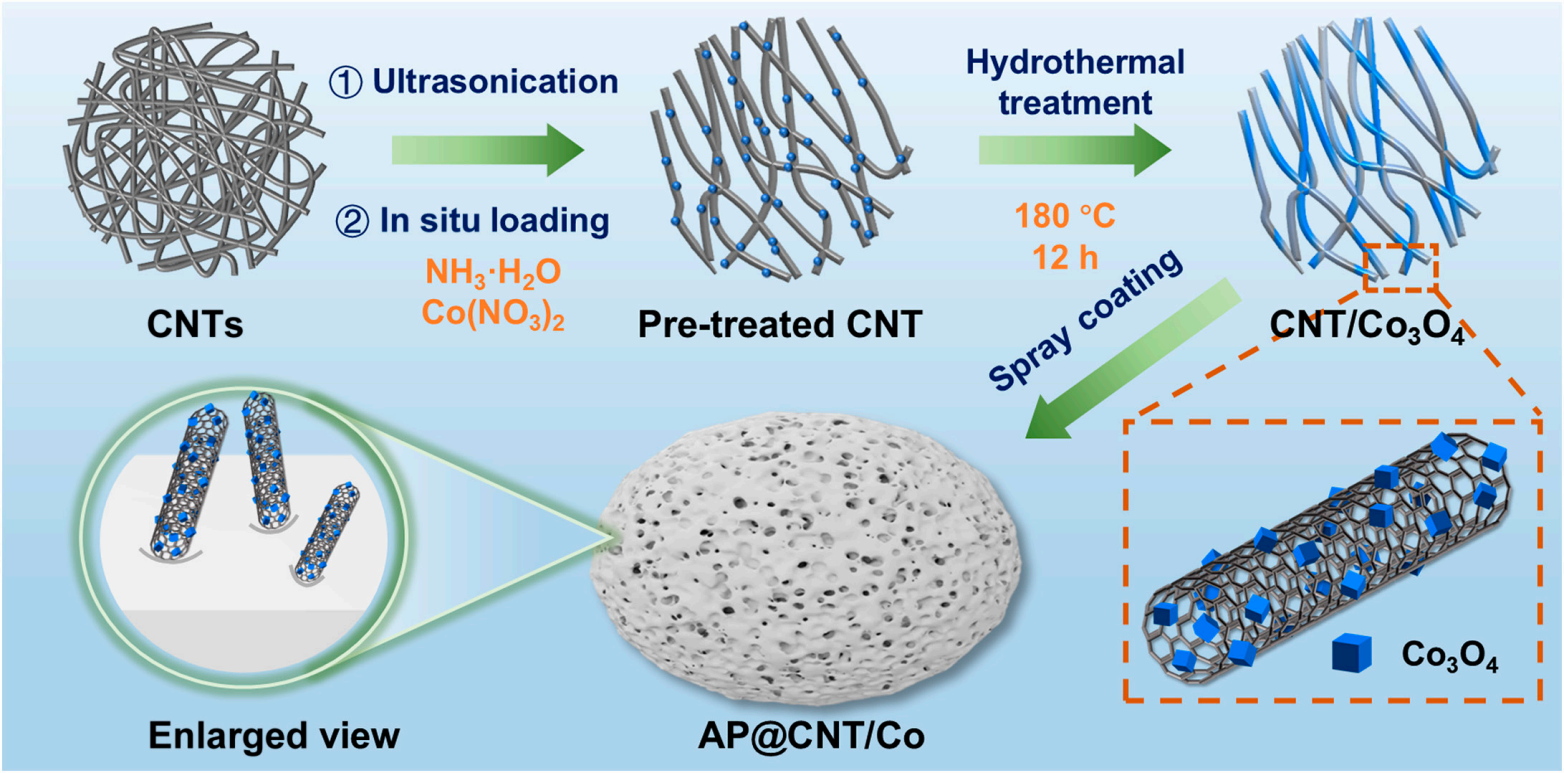
Schematic illustration for fabricating heterostructured CNT/Co_3_O_4_ and AP@CNT/Co composites. CNT, carbon nanotube; AP, ammonium perchlorate.

Inspired by these developments, this study proposes a synergistic strategy that integrates engineered thermal conduction pathways with precision catalysis to achieve high-efficiency energy release from AP. It is expected that constructing heterostructured thermal conduits on the AP surface can concurrently facilitate heat transfer and catalytic decomposition. Herein, the in situ growth of catalytically active Co_3_O_4_ nanoclusters on highly conductive CNTs was realized to form heterostructured CNT/Co_3_O_4_. It was then precisely embedded onto the AP surface via a spray drying-suspension coating technology, creating integrated AP@CNT/Co composites (as illustrated in Fig. 1). Within this architecture, the CNTs serve as rapid thermal conduction pathways, while the Co_3_O_4_ nanoclusters provide catalytic sites for proton/electron transfer and promote the oxidation of gaseous products. Furthermore, the resulting surface microporosity increases the specific surface area and enhances mass diffusion. Crucially, the retained large-particle morphology of pristine AP avoids the practical drawbacks associated with ultrafine powders, such as high sensitivity, complex processing, and pronounced hygroscopicity. This synergistic strategy is anticipated to enable focused, rapid, and efficient energy release from AP, offering a generic interfacial engineering paradigm for advanced energetic composites.

## Results and Discussion

### Characterizations of heterostructured CNT/Co_3_O_4_

**Fig. 2.**
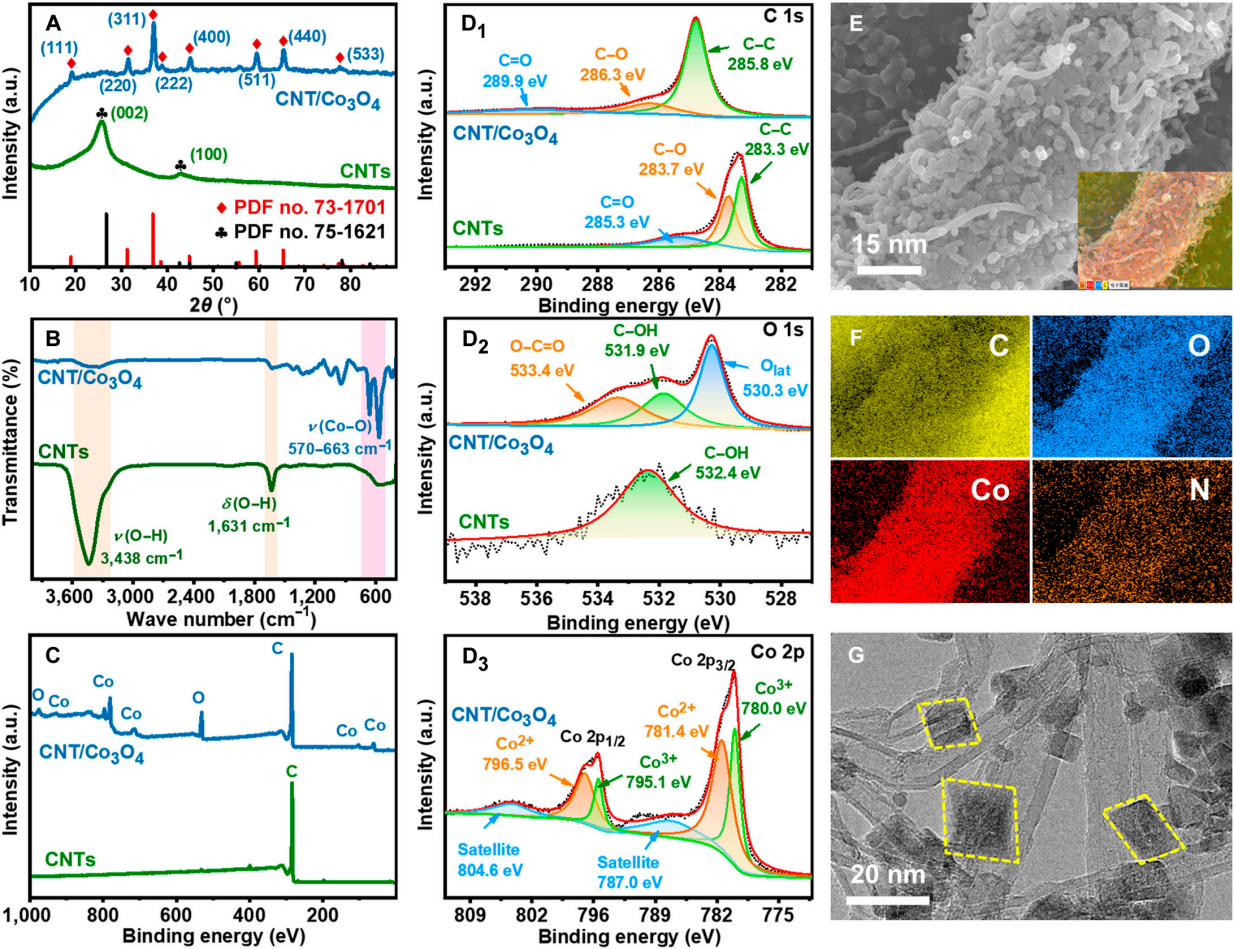
Structural characterization results for CNTs and heterostructured CNT/Co_3_O_4_: (A) x-ray diffraction (XRD) patterns, (B) Fourier transform infrared (FT-IR) spectra, (C) x-ray photoelectron spectroscopy (XPS) survey spectra, and high-resolution XPS spectra of (D_1_) C 1s, (D_2_) O 1s, and (D_3_) Co 2p; (E) scanning electron microscopy (SEM), (F) energy-dispersive spectroscopy (EDS), and (G) transmission electron microscopy (TEM) images of heterostructured CNT/Co_3_O_4_.

To confirm the in situ deposition of Co_3_O_4_ onto CNT surface, the chemical structure and morphology of the starting materials and synthesized samples were characterized, with the results presented in Fig. 2 and Fig. S1. Figure 2A displays the x-ray diffraction (XRD) patterns of pristine CNTs and heterostructured CNT/Co_3_O_4_. The CNTs exhibit a prominent broad diffraction peak at ~26°, indexed to the (002) crystallographic plane (PDF no. 75-1621), which is characteristic of multiwalled CNTs and indicative of a well-developed graphitic structure. For heterostructured CNT/Co_3_O_4_, while retaining the characteristic (002) peak of CNTs with a reduced intensity, a series of new, sharp diffraction peaks emerge at 2*θ* = 19.1°, 31.3°, 36.9°, 44.8°, 59.4°, and 65.2°. These peaks exhibit excellent correspondence with the standard pattern for cubic spinel Co_3_O_4_ (PDF no. 73-1701), being assigned to the (111), (220), (311), (400), (511), and (440) planes, respectively. The well-defined peaks and precise peak positions confirm the successful deposition of highly crystalline, cubic spinel-structured Co_3_O_4_ nanoclusters onto the CNT surface, consistent with previous findings [32]. The Fourier transform infrared (FT-IR) spectra of heterostructured CNT/Co_3_O_4_ are presented in Fig. 2B. The broad absorption bands observed at 3,438 and 1,631 cm^−1^ are attributed to the O–H stretching vibration (*ν*(O–H)) and bending vibration (*δ*(O–H)) of adsorbed water molecules on the CNT surface. Crucially, for heterostructured CNT/Co_3_O_4_, distinct absorption bands are evident in the low-wave-number region (570 to 663 cm^−1^). These bands correspond to the characteristic Co–O stretching vibrations (*ν*(Co–O)) within the spinel Co_3_O_4_ lattice, providing further confirmation of the successful deposition of Co_3_O_4_.

To gain deeper insights into the chemical structure and interfacial characteristics between CNTs and Co_3_O_4_, x-ray photoelectron spectroscopy (XPS) analysis was conducted with results depicted in Fig. 2C and D. The XPS survey spectrum of heterostructured CNT/Co_3_O_4_ Fig. 2C) confirms the presence of Co, O, and C elemental signals. High-resolution spectra of the C 1s, O 1s, and Co 2p core levels provide detailed information on chemical states and interfacial properties. The high-resolution C 1s spectrum (Fig. 2D_1_) is deconvoluted into 3 characteristic peaks centered at 285.8, 286.3, and 289.9 eV, assigned to C–C, C–O, and C=O bonds, respectively [33]. The high-resolution O 1s spectrum (Fig. 2D_2_) is fitted with 3 components at binding energies of 530.3, 531.9, and 533.4 eV, corresponding to lattice oxygen (O_lat_), C–O, and C=O bonds, respectively [34]. The high-resolution Co 2p spectrum (Fig. 2D_3_) reveals the valence states of Co^2+^ and Co^3+^. The spectrum exhibits characteristic Co 2p_3/2_ and Co 2p_1/2_ spin–orbit doublets. Deconvolution yields peaks at 780.0 and 795.1 eV for Co^3+^ and peaks at 781.4 and 796.5 eV for Co^2+^. The characteristic satellite peak structure further confirms that the synthesized material conforms to the structural features of Co_3_O_4_. Moreover, the binding energy difference between the Co 2p_3/2_ and Co 2p_1/2_ peaks is approximately 15 eV, which is highly consistent with that reported for cubic spinel Co_3_O_4_, confirming the high phase purity of the in situ deposited Co_3_O_4_.

The surface morphology and internal architecture of the heterostructured CNT/Co_3_O_4_ were elucidated using scanning electron microscopy (SEM) and transmission electron microscopy (TEM), as depicted in Fig. 2E to G. Comparative analysis with Fig. S1a reveals that the in situ grown Co_3_O_4_ nanoclusters form a continuous coating over the CNT surface. The spatial homogeneity of this coating is corroborated by the uniform distribution of the Co and O elements observed in the elemental mapping results (Fig. 2F). The minor presence of N originates from the ammonia solution utilized during synthesis. Furthermore, the nanoscale deposition of Co_3_O_4_ onto the CNT surface is directly visualized in the TEM micrographs. Figure 2G distinctly reveals cubic Co_3_O_4_ nanocrystals with edge lengths on the order of tens of nanometers, exhibiting well-defined facets and sharp crystalline contours, indicative of their high degree of crystallinity. These morphological observations are in agreement with the crystallographic data obtained from XRD analysis. In summary, these results demonstrate that CNTs serve as an effective scaffold for the heterogeneous nucleation and high-crystallinity growth of Co_3_O_4_ nanoclusters, conclusively confirming the successful fabrication of heterostructured CNT/Co_3_O_4_ via the hydrothermal method.

### Characterizations of AP@CNT/Co composites

**Fig. 3.**
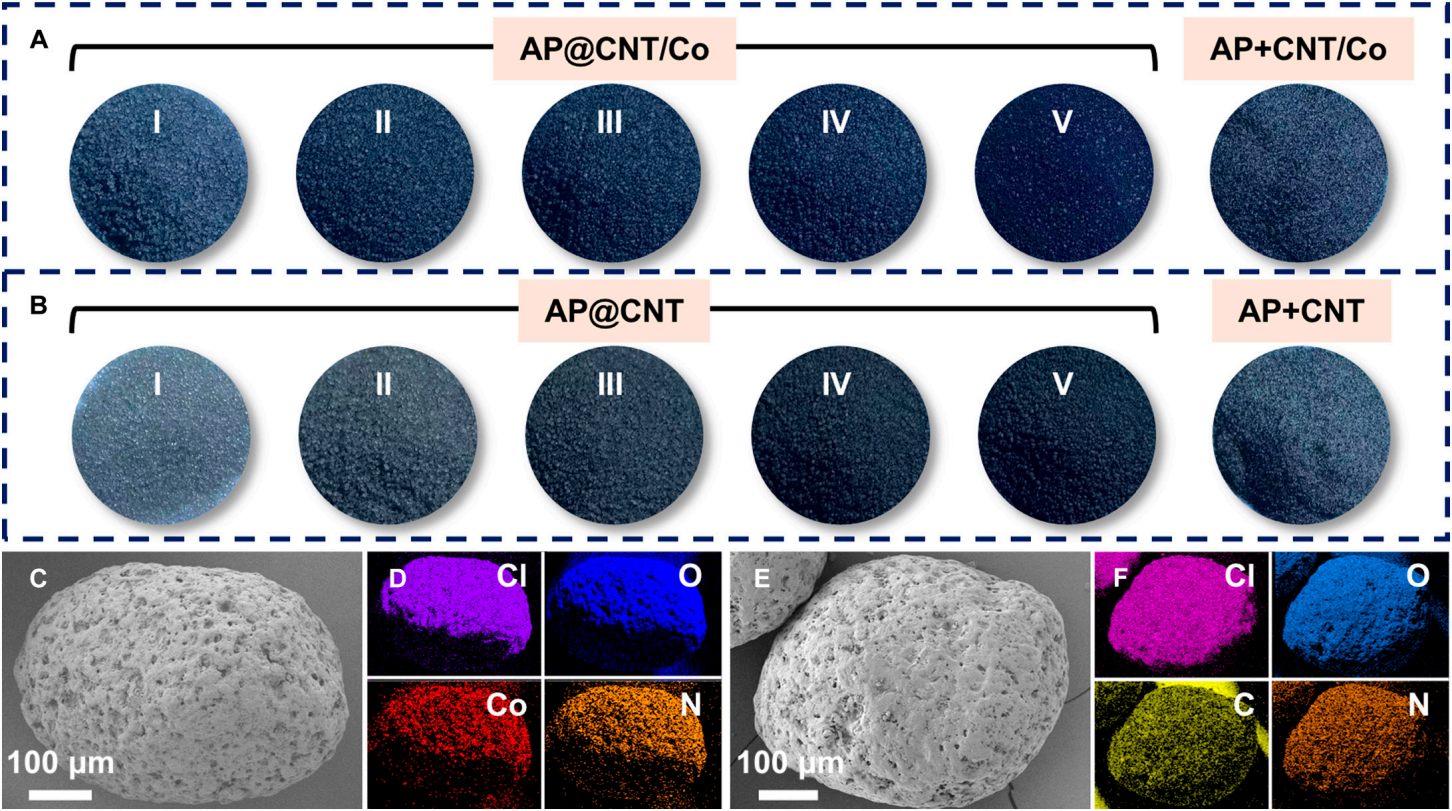
(A) Comparative photographs of AP@CNT/Co composites sampled at equivalent time intervals and the physical mixture AP+CNT/Co; (B) comparative photographs of AP@CNT composites sampled at equivalent time intervals and the physical mixture AP+CNT; (C) SEM image and (D) EDS mapping of the AP@CNT/Co composites; (E) SEM image and (F) EDS mapping of the AP@CNT composites.

To elucidate the embedding of CNTs and heterostructured CNT/Co_3_O_4_ onto the AP surface, the prepared samples were extracted at equivalent time intervals during processing (denoted as I to V, where V represents the final product) for visual inspection. Their appearance was compared with physical mixtures of identical composition (AP+CNT/Co and AP+CNT), as presented in Fig. 3 and Figs. S2 and S3.

It is evident that progressive darkening of the samples occurs with continued embedding of CNTs and CNT/Co_3_O_4_ onto the AP surface, indicative of the gradual accumulation of these components. The AP@CNT/Co and AP@CNT composites exhibit uniform coloration, signifying the homogeneous distribution of CNTs and CNT/Co_3_O_4_ across the AP surface. In contrast, the physically mixing counterparts (AP+CNT/Co and AP+CNT) display inadequate homogeneity, visually demonstrating the inherent limitation of physical mixing in achieving uniform integration between catalysts and targets. This limitation underscores the distinct advantage of the precision catalysis strategy employed in this work. Compared to pristine AP (Fig. S1c), the synthesized AP@CNT and AP@CNT/Co composites feature a porous surface morphology, resulting from water droplet etching during processing. This microporous architecture effectively enhances the specific surface area of the composites, facilitating the provision of abundant reactive sites and thereby augmenting energy release efficiency. Furthermore, elemental mapping analysis and high-magnification views of the AP@CNT and AP@CNT/Co surfaces confirm the existence of CNTs and CNT/Co_3_O_4_ on the AP surface.

**Fig. 4.**
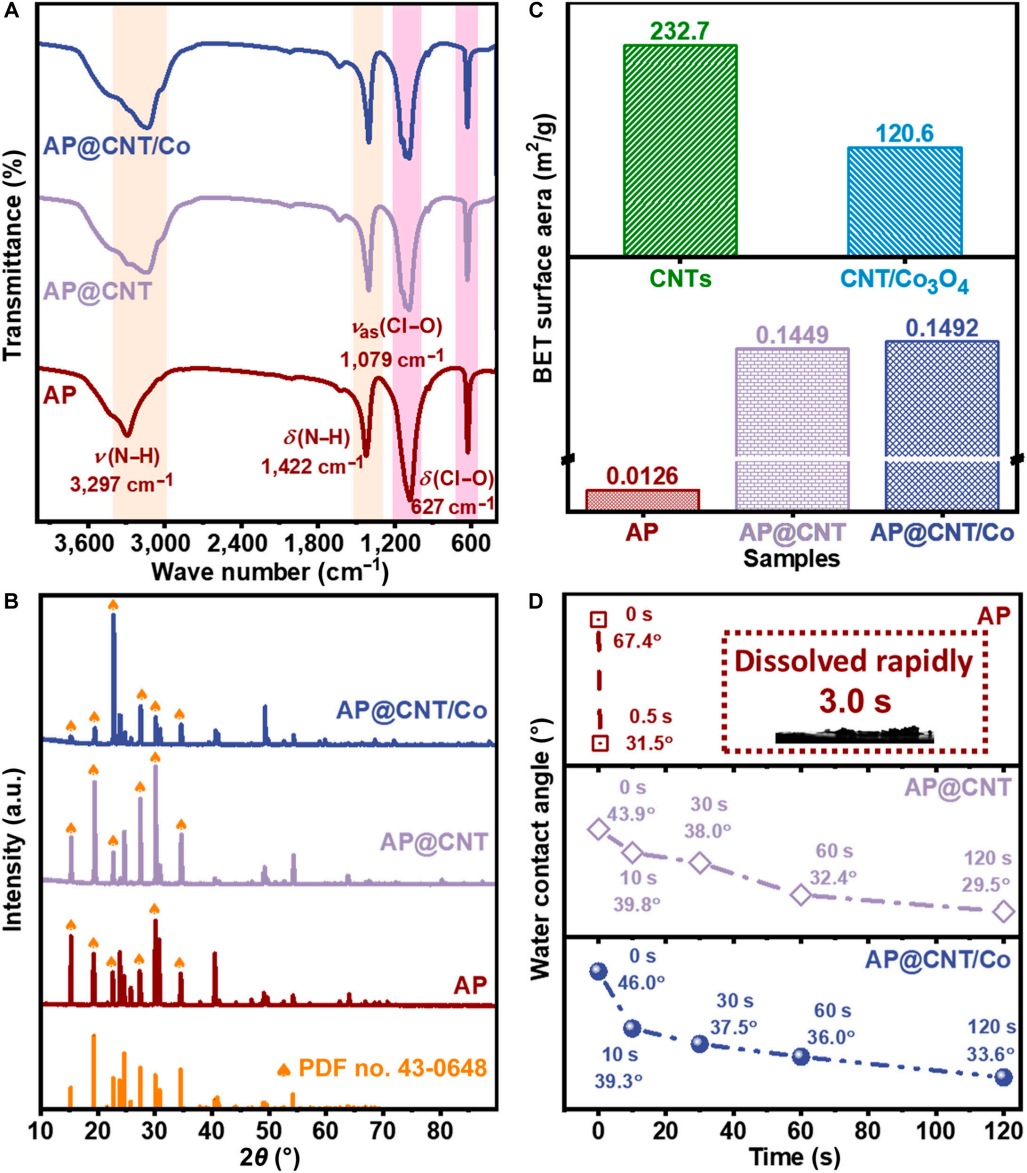
Comparison of (A) FT-IR spectra, (B) XRD patterns, (C) Brunauer–Emmett–Teller (BET) surface aeras, and (D) water contact angle measurement results for AP, AP@CNT, and AP@CNT/Co composites.

The structural characterization results for AP, AP@CNT, and AP@CNT/Co composites are presented in Fig. 4. The FT-IR spectra (Fig. 4A) reveal 2 prominent characteristic peaks for pristine AP at 3,297 and 1,422 cm^−1^, assigned to N–H stretching (*ν*(N–H)) and bending vibrations (*δ*(N–H)), respectively. Additional AP signatures at 1,079 and 627 cm^−1^ originate from Cl–O asymmetric stretching (*ν*_as_(Cl–O)) and bending vibrations (*δ*(Cl–O)), respectively. The FT-IR profiles of AP@CNT and AP@CNT/Co composites align with that of pure AP, indicating that minor embedding of CNTs and heterostructured CNT/Co_3_O_4_ does not alter the intrinsic chemical structure of AP. Notably, the characteristic AP peak at 3,297 cm^−1^ undergoes a slight redshift in both composites.

For AP@CNT, this redshift is attributed to cation–π interactions between the π-electron cloud in CNTs and NH_4_^+^ in AP [35]. For AP@CNT/Co, where CNT surfaces are coated with Co_3_O_4_, the redshift primarily reflects interactions between Co_3_O_4_ and NH_4_^+^, potentially mediated through electrostatic adsorption, hydrogen bonding, or surface coordination [36,37]. These interactions slightly weaken N–H bond strength, manifesting as peak broadening and redshift. The observed N–H redshift signifies chemical interactions between the embedded materials and AP beyond mere physical embedding. Given the minor extent of redshift and low embedded content (<1 wt%), the AP chemical structure remains unperturbed. However, these interactions may reduce the thermal stability of AP, as will be validated subsequently via thermal analysis.

XRD patterns (Fig. 4B) confirm that the crystalline phase of AP was preserved after embedding CNTs or CNT/Co_3_O_4_, primarily influencing the preferential orientation and relative exposure of specific crystallographic planes. Brunauer–Emmett–Teller surface area analysis (Fig. 4C) reveals a 112.1 m^2^ g^−1^ (48.2%) reduction for CNT/Co_3_O_4_ versus CNTs, evidencing dense Co_3_O_4_ deposition on CNTs. Both AP@CNT and AP@CNT/Co composites exhibit an over order-of-magnitude increase in specific surface area compared to pure AP, which stems from both the embedded carbon nanostructures and the microporous architecture formed. The elevated specific surface area can provide potential reactive channels and energy release efficiency in return.

Water contact angle measurements (Fig. 4D and Fig. S4) were further conducted to assess the effect of embedding. The complete video records for droplet evolution on the surfaces can be found in Video S1. Different wetting behaviors are observed. Water droplets on AP exhibit rapid absorption, with contact angles abruptly reduced by over 50% within 0.5 s and complete disappearance within 3 s, reflecting the pronounced hygroscopicity of AP. In contrast, AP@CNT and AP@CNT/Co composites demonstrate significantly retarded absorption, maintaining contact angles of 29.5° and 33.6° after 120 s, respectively. This confirms that embedding CNTs or CNT/Co_3_O_4_ effectively improves the hygroscopicity of AP, conducive to long-term storage stability.

### Thermal decomposition behaviors

**Fig. 5.**
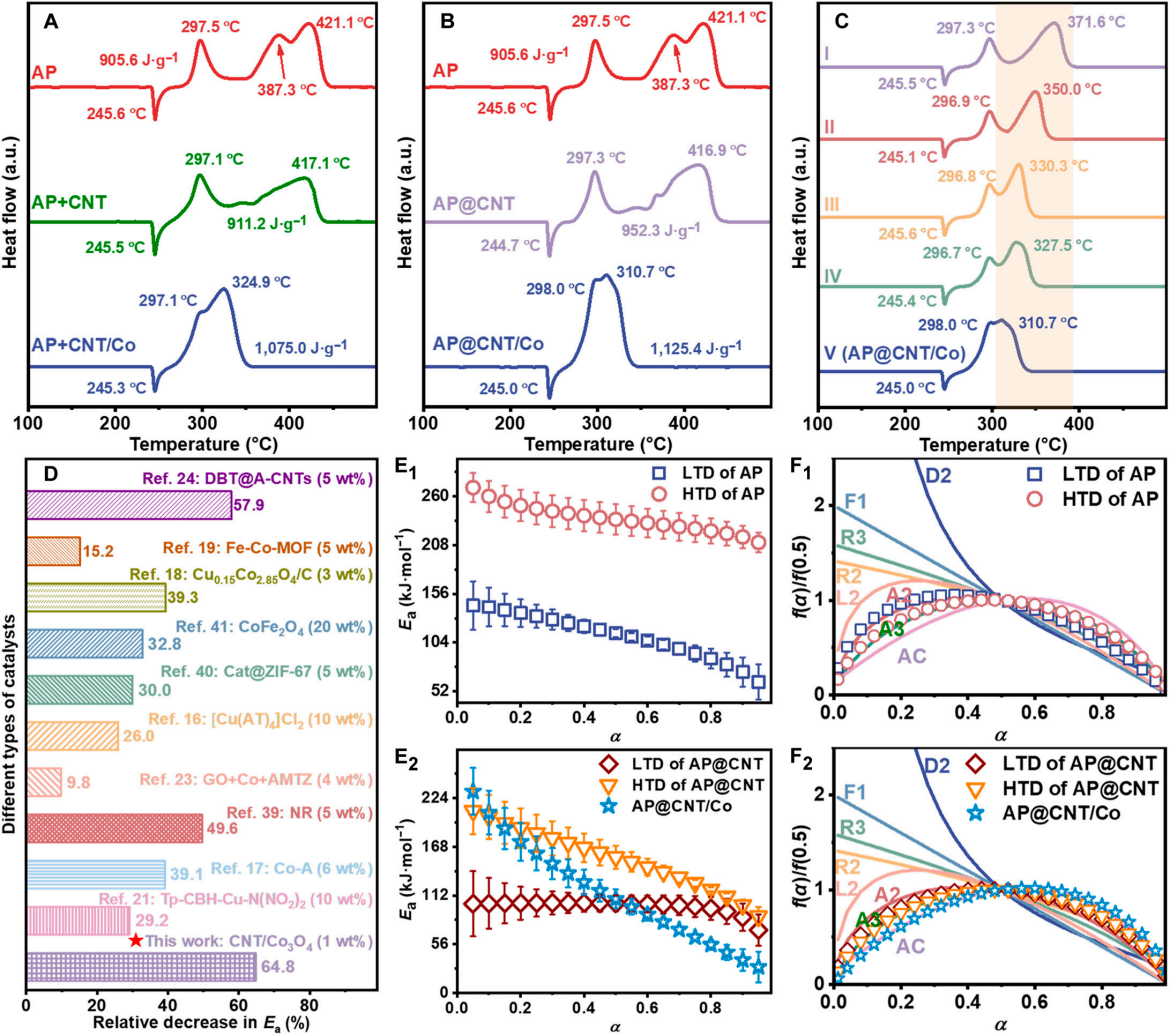
Differential scanning calorimetry (DSC) profiles of as-prepared samples: (A) AP, AP+CNT, and AP+CNT/Co; and (B) AP, AP@CNT, and AP@CNT/Co; (C) AP@CNT/Co composites sampled at equivalent time intervals; (D) comparative reduction in thermal decomposition *E*_a_ of AP by different catalysts; (E_1_ and E_2_) the *E*_a_ dependence on *α* profiles derived via the Friedman method; (F_1_ and F_2_) kinetic models determined by the combined kinetic analysis (CKA) method. DBT, dibutyltin dichloride; MOF, metal–organic framework; Cat, catocene; GO, graphene oxide; AMTZ, 3-amino-1,2,4-triazole; NR, neutral red; Co-A, cobalt alginate; Tp-CBH, triformyl phloroglucinol-carbohydrazide; LTD, low-temperature decomposition; HTD, high-temperature decomposition.

The thermal decomposition behavior and associated thermodynamic parameters of the synthesized samples were tested via simultaneous differential scanning calorimetry/thermogravimetric analysis (DSC/TG), with the results presented in Fig. 5 and Fig. S5. Pristine AP exhibits one endothermic event and 2 exothermic decomposition processes within the tested temperature range. The endothermic peak near 245.6 °C corresponds to the orthorhombic-to-cubic phase transition of AP crystals, while exothermic peaks between 280 and 450 °C reflect its multistep decomposition, evidenced by the 2-stage mass loss in TG curves. The proton transfer occurred during the LTD process around ~297.5 °C, generating NH_3_ and HClO_4_, while subsequent HTD entails their gas-phase reactions and HClO_4_ pyrolysis [38]. It can be found that the embedding materials and interfacial contact play an important role in the thermal decomposition behaviors for AP. For the physical mixture AP+CNT (Fig. 5A), the incorporation of CNTs minimally affects the decomposition of AP, only slightly reducing the peak temperature for the HTD process. Mixing with CNT/Co_3_O_4_ significantly lowers the peak temperature for HTD by 96.2 °C, transforming the decomposition of AP+CNT/Co into one consecutive exothermic stage.

The advantage of the precision catalysis strategy becomes evident upon embedding CNT/Co_3_O_4_ onto the AP surface (Fig. 5B). While AP@CNT shows no significant HTD peak shift versus AP+CNT, its decomposition heat release increases by 41.1 J g^−1^. The AP@CNT/Co composite exhibits a further reduced HTD peak temperature of 310.7 °C, with a mere 12.7 °C separation between LTD and HTD peaks, 110.9 and 15.1 °C narrower than those of AP and AP+CNT/Co, respectively. Moreover, AP@CNT/Co achieves an increased heat release of 1,125.4 J g^−1^, a 24.3% enhancement relative to that of pristine AP. The maximum mass loss rate derived from TG–DTG curves reflects the reaction rate of samples. Under precision catalysis, AP@CNT/Co attains a maximum mass loss rate of 2.5% °C^−1^, representing a 31.6% increase over that of AP and outperforming those of all physically mixing counterparts. In addition, these results demonstrate the superior catalytic efficacy of Co_3_O_4_ over CNTs for AP decomposition, with the AP@CNT/Co composite enabling focused exothermicity and enhanced heat release efficiency. The DSC profiles of AP@CNT/Co composites sampled at equivalent time intervals (Fig. 5C) reveal continuous HTD peak temperature reduction with increasing CNT/Co_3_O_4_ embedding. Notably, even sample I (lowest loading) exhibits a lower HTD peak temperature than AP and AP@CNT, further verifying the inherent advantage of the precision catalysis.

### Nonisothermal decomposition kinetics

To investigate the decomposition kinetics for the synthesized samples, their kinetic parameters were calculated using the Kissinger method, Friedman method, and combined kinetic analysis (CKA) approach, with the results summarized in Table S1 and Fig. 5D to F. For pristine AP, the calculated *E*_a_ by the Kissinger method was 116.3 kJ mol^−1^ for LTD and 240.9 kJ mol^−1^ for HTD, indicating a substantial energy barrier of 124.6 kJ mol^−1^ between the 2 decomposition stages. Embedding CNTs or CNT/Co_3_O_4_ onto the AP surface significantly reduced *E*_a_ in both AP@CNT and AP@CNT/Co composites, particularly for the HTD stage, demonstrating that precision catalysis offers higher catalytic efficiency and effectively lowers the decomposition energy barriers of AP. Compared to pristine AP, the AP@CNT composite shows reductions in *E*_a_ of 7.0% and 30.1% for LTD and HTD, respectively, suggesting the role of CNTs in facilitating thermal transport and markedly reducing the HTD energy barrier. In contrast, heterostructured CNT/Co_3_O_4_ exhibited superior catalytic performance: beyond merging multistage decomposition of AP into a single, concentrated exothermic stage, it reduced the overall *E*_a_ to 125.6 kJ mol^−1^, a substantial reduction of 231.6 kJ mol^−1^ (−64.8%) compared to that of pristine AP.

A comparison of the *E*_a_ reduction efficiency of various catalysts [16–19,21,23,24,39–41] is displayed in Fig. 5D, with their respective loadings annotated. Most prior studies evaluated catalysts via physically mixing them with AP. Such macroscopic mixing often fails to ensure intimate microscopic contact, typically necessitating high catalyst loadings (≥5 wt%). However, excessive catalyst incorporation risks diminished energy density, chemical incompatibility, and mechanical property degradation in practical applications. The precision catalysis strategy employed herein achieves substantial promotion of AP decomposition at extremely lower catalyst loadings. Notably, this strategy delivers the most pronounced *E*_a_ reduction among the cited examples.

The evolution of *E*_a_ with conversion rate (*α*) is depicted in Fig. 5E. Pristine AP exhibits gradually declining *E*_a_ values during decomposition, averaging 113.1 kJ mol^−1^ for LTD and 235.8 kJ mol^−1^ for HTD. For AP@CNT, the *E*_a_ for LTD shows minimal *α* dependence with a reduced value compared to that of pristine AP, while the *E*_a_ for HTD decreases markedly with *α*, signifying reduced energy barriers at elevated temperatures. The AP@CNT/Co composite exhibits a further reduced average *E*_a_ of 110.5 kJ mol^−1^ and a steeper negative slope in the *E*_a_–*α* profile, indicating progressively accelerated decomposition kinetics.

The most appropriate physical models governing the decomposition of samples were identified via the combined kinetic analysis method (Fig. 5F_1_ and F_2_). The kinetic models for AP decomposition in the composites differ fundamentally from that of pristine AP. The decomposition of pristine AP follows 2D (A2) and 3D (A3) random nucleation and growth model for LTD and HTD, respectively. The embedding of CNTs shifts both the LTD and HTD stages toward the A3 model, suggesting enhanced reaction dimensionality and facilitated decomposition. Remarkably, introducing CNT/Co_3_O_4_ transforms the mechanism into an autocatalytic model. This transformation reflects an increase in the reaction freedom for AP, whose decomposition is also catalyzed by its own products. The autocatalytic model often implies accelerated decomposition and lower energy barriers, consistent with the *E*_a_ dependency on *α* obtained by the Friedman method. This shift arises from strong interfacial interactions between Co_3_O_4_ and AP, particularly the involvement of unsaturated 3d orbitals of Co species in promoting secondary reactions of decomposition intermediates [41,42], thereby rendering the decomposition highly susceptible to Co_3_O_4_.

### Decomposition path analysis

The thermal decomposition mechanism of AP has been investigated through analysis of its gaseous products under varying conditions. While experimental parameters influence product distribution, decomposition predominantly yields N*_x_*O*_y_*, N_2_, Cl_2_, H_2_O, and trace HCl [38], with nitrogen oxides comprising NO, NO_2_, and N_2_O (Eq. (1)). This process involves 2 primary steps: initial proton transfer from cations to anions, generating NH_3_ and HClO_4_ (Eq. (2)), followed by complex reactions among intermediates and radicals. Variations in the relative abundance of nitrogen oxides with different oxidation states reflect the catalytic influence on decomposition pathways, as catalysts can alter the yields of nitrogen oxides by governing electron/proton transfer and redox reactions.

$$NH_{4}\text{Cl}O_{4}=N_{x}O_{y}+N_{2}+{\text{Cl}}_{2}+H_{2}O+\text{HCl}$$ (1)

$$\mathrm{Cl}{O_{4}}^{-}+N{H_{4}}^{+}=NH_{3}+\text{HCl}O_{4}$$ (2)

**Fig. 6.**
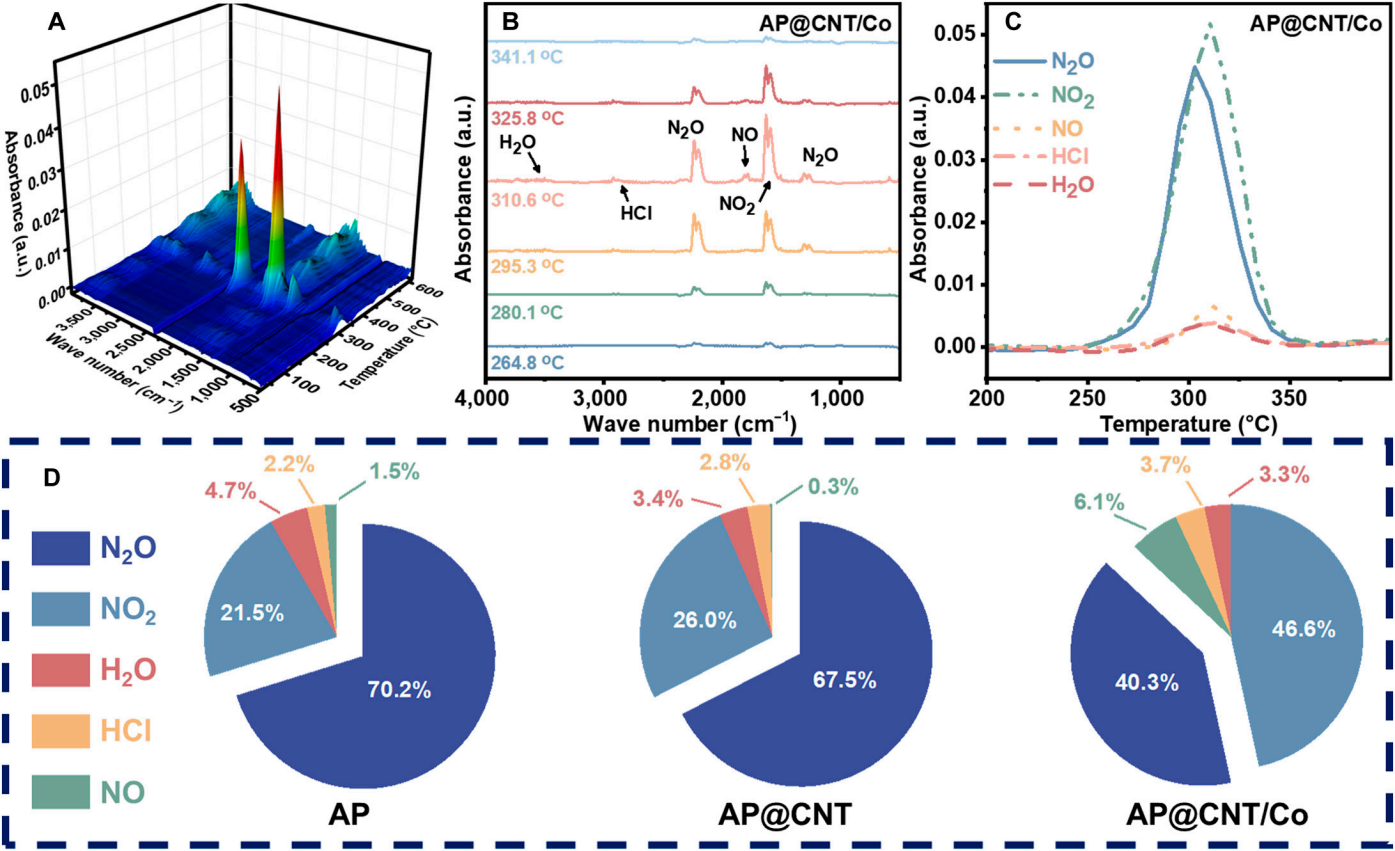
FT-IR analysis of gaseous products for the decomposition of samples: (A) full-range spectra; (B) spectra near decomposition peak temperatures; (C) temperature-dependent profiles of typical nitrogen oxides for AP@CNT/Co; (D) comparative relative content of typical nitrogen oxides.

The composition of gaseous products was analyzed using FT-IR spectroscopy, with the results shown in Fig. 6 and Figs. S6 and S7. The full-range FT-IR spectra of gaseous products reveal 2 distinct product releasing stages for both AP and AP@CNT with increasing temperature, whereas AP@CNT/Co exhibits a single, concentrated release near 300 °C, consistent with their respective thermal decomposition profiles. Representative FT-IR spectra acquired near the decomposition peak temperatures (Fig. S7a and b, using AP as an example) identify major gaseous products including N_2_O, NO_2_, and trace amounts of NO, HCl, and H_2_O. AP@CNT and AP@CNT/Co yield identical product species, albeit with differing concentrations as discerned from spectral intensities.

Nitrogen oxides N_2_O, NO_2_, and NO were selected as 3 typical gaseous products to elucidate species distribution across decomposition stages and the oxidation reaction occurred (Fig. 6D and Fig. S7c and f). N_2_O dominates the product profile for both AP (70.2%) and AP@CNT (67.5%), indicating limited oxidation for nitrogen oxidation. NO_2_ is negligible during LTD but increases substantially in HTD, signifying enhanced nitrogen oxidation at higher temperatures. As for AP@CNT/Co, higher oxidation state species NO_2_ and NO become predominant with relative contents of 46.6% and 6.1%, respectively. This shift is attributed to the pronounced oxidative capacity of heterostructured CNT/Co_3_O_4_, promoting the release of species with a higher oxidation state. This phenomenon was also proven in the catalytic decomposition of AP by Co_3_O_4_@ZnO [43]. Mechanistically, elevated NO_2_ levels may arise from (a) direct oxidation of NH_3_ to higher nitrogen oxides by Co^3+^/Co^2+^ in Co_3_O_4_: while NH_3_ can be oxidized to N_2_ under certain conditions [44], the low Co_3_O_4_ loading and insufficient density of active sites in the composite may lead to incomplete oxidation, allowing NO accumulation and its subsequent oxidation to NO_2_ under oxygen-rich conditions; (b) Co_3_O_4_-catalyzed decomposition of perchlorate anions generating reactive oxygen species, which further oxidize reduced nitrogen intermediates to NO_2_. A detailed mechanism will be discussed later.

### Combustion performance tests

**Fig. 7.**
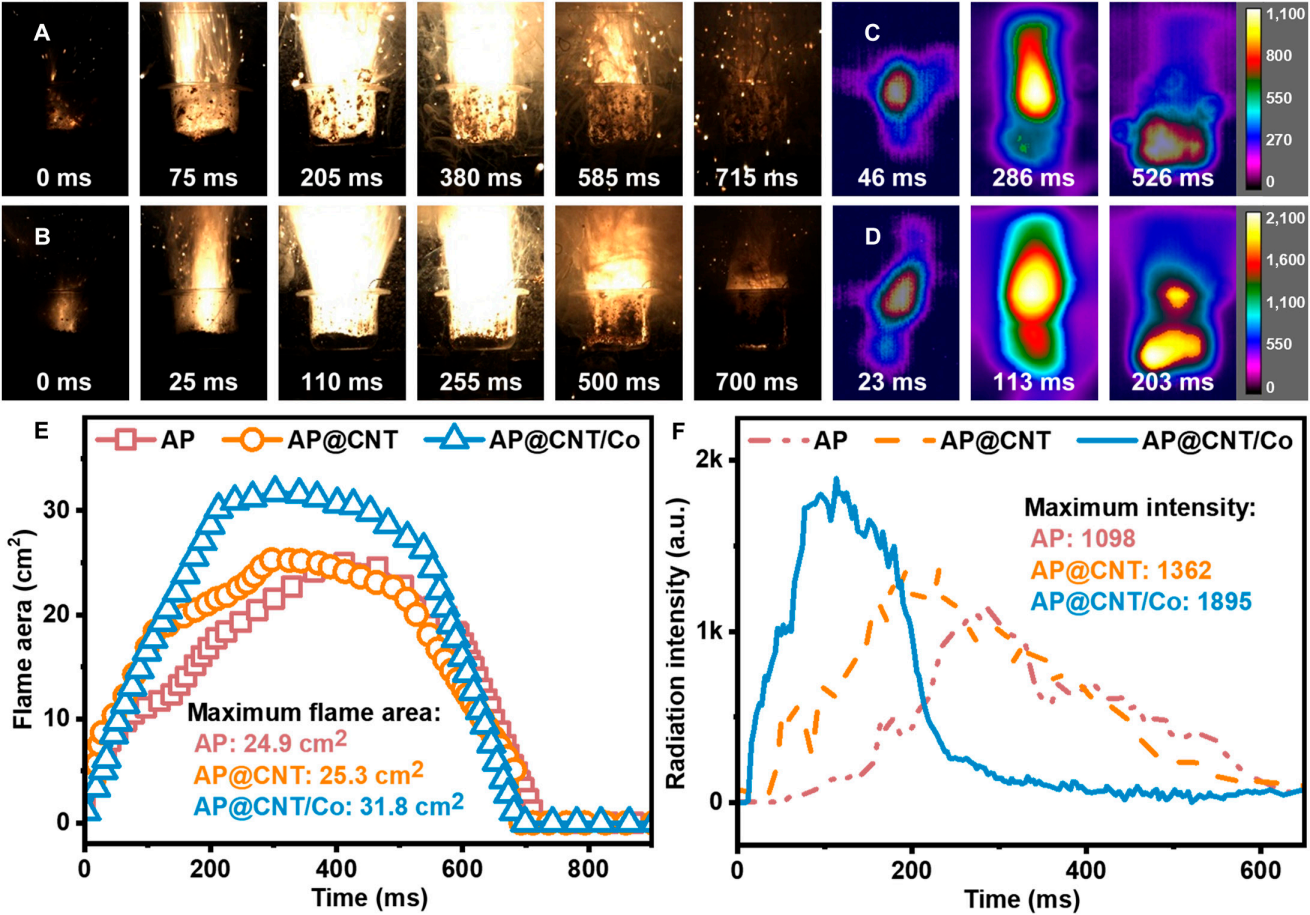
Flame sequences and infrared radiation images of mixtures with equal-mass Al powders: (A and C) AP and (B and D) AP@CNT/Co; (E) flame area evolution and (F) infrared radiation intensity profiles.

Thermal decomposition studies of the synthesized AP@CNT/Co composite under slow heating conditions confirmed its enhanced energy release efficiency. To further evaluate energy release under rapid combustion, the composite was uniformly mixed with an equal mass of aluminum (Al) powder, enabling combustion and acquisition of flame sequence photography and infrared radiation intensity. For comparison, combustion of pristine AP and AP@CNT mixed equivalently with Al powder was assessed identically (Fig. 7 and Fig. S8). The complete combustion process can be found in Video S2.

The combustion sequence images displayed in Fig. 7A and B and Fig. S8a reveal that the AP@CNT/Co exhibits the fastest combustion rate, achieving complete reaction within 700 ms, the shortest duration observed. From the perspective of flame structure, AP@CNT and AP@CNT/Co produce brighter flames with broader combustion envelopes, indicative of more vigorous reactions. Notably, numerous luminous agglomerates are visible surrounding the flames, corresponding to molten Al combustion products. Analogous to Al agglomeration on the propellant burning surface, fewer or smaller agglomerates signify higher combustion efficiency [45]. It can be seen that pristine AP generates more numerous and larger agglomerates, while AP@CNT and AP@CNT/Co exhibit obviously reduced Al agglomeration above the flame, implying that greater energy release facilitates rapid and efficient Al oxidation. Flame area profiles (Fig. 7E) demonstrate that AP@CNT/Co achieves the most rapid expansion after being ignited, sustaining the largest flame area of 31.8 cm^2^, 27.7% and 25.7% greater than those of AP and AP@CNT, respectively. The increase is directly related to the improvement of heat distribution by the thermal conductivity effect of CNTs and the strengthening of the gas-phase reaction promoted by Co_3_O_4_, which corresponds to the test results of rapid release of gaseous products and high energy release efficiency.

The flame infrared radiation profiles in Fig. 7C and D and Fig. S8b were captured by a high-speed infrared camera, and intensity evolution was obtained as shown in Fig. 7F. Throughout the whole combustion, AP@CNT/Co exhibits the highest infrared radiation intensity, peaking at 1,895, representing 72.6% and 39.1% increases over those of AP and AP@CNT, respectively. Its radiation intensity rises most rapidly with a steeper gradient after being ignited, indicating faster reaction rates and more heat release. This enhancement stems from elevated combustion temperatures and more efficient energy release, aligning with the decomposition catalytic mechanism by heterostructured CNT/Co_3_O_4_ for perchlorate intermediates. These results demonstrate that CNT and Co_3_O_4_ nanoclusters synergistically optimize the exothermic pathways of AP, thereby accelerating its decomposition and enhancing its combustion efficiency.

### Mechanism investigation on the energy release of AP@CNT/Co composites

**Fig. 8.**
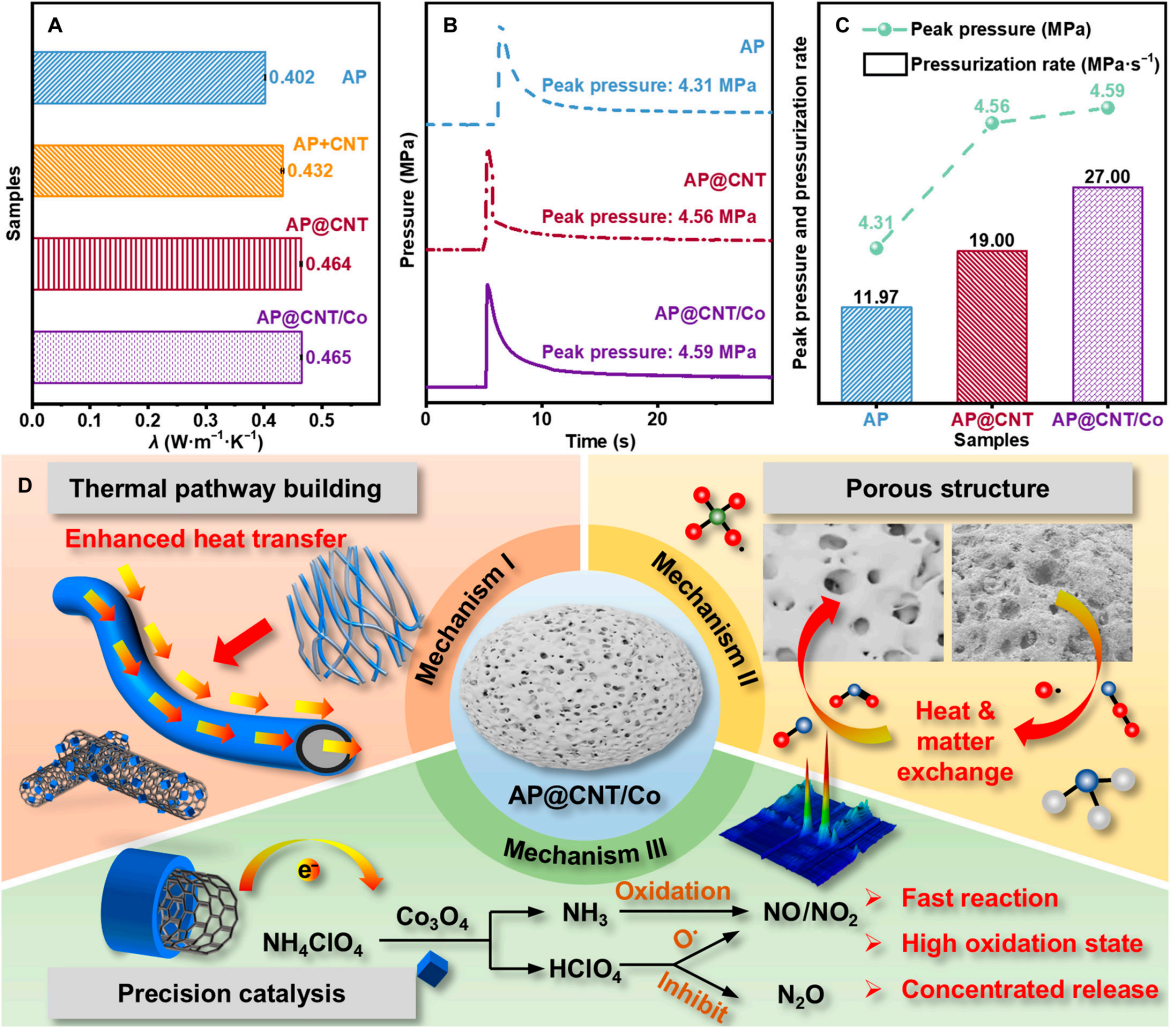
(A) Thermal conductivity and (B and C) pressurization rate profiles; (D) proposed mechanism of the enhanced energy release of the AP@CNT/Co composite.

Both thermal decomposition and combustion performance tests demonstrated that embedding heterostructured CNT/Co_3_O_4_ onto the AP surface enhances its energy release efficiency. To elucidate the underlying mechanisms, the thermal conductivity (*λ*) and pressurization rate of the samples were characterized, as shown in Fig. 8A to C. Thermal conductivity serves as a critical indicator of internal heat transfer efficiency. Notably, *λ* measurements were performed on cylindrical pellets of the samples (Fig. S9). They show that the *λ* value of the system is profoundly influenced by the catalyst type and interfacial contact. Pristine AP exhibits a *λ* of 0.402 W m^−1^ K^−1^, and it increases to 0.432 W m^−1^ K^−1^ for the AP+CNT mixture, attributable to the exceptional thermal conductivity of CNTs. Embedding CNTs onto the AP surface (AP@CNT) further elevates *λ* to 0.464 W m^−1^ K^−1^, representing 15.4% and 7.4% enhancements over those of pristine AP and AP+CNT, respectively. The lower *λ* of AP+CNT versus that of AP@CNT underscores the inherent limitation of mechanical mixing in achieving uniform dispersion at the microscale, corroborated by the nonuniform coloration of AP+CNT pellets. This validates the distinct advantage of the precision catalysis strategy in optimizing catalyst dispersion and homogeneity. AP@CNT/Co exhibits a *λ* comparable to that of AP@CNT, indicating that thermal enhancement primarily stems from CNTs, with less contribution from Co_3_O_4_ nanoclusters.

Pressurization rate tests (Fig. 8B and C) were carried out to evaluate gas release and evolution kinetics. After being ignited, the pressure rapidly ascends to a peak before gradual decline. The peak pressure reflects the total gas yield, while the pressurization rate (slope of the pressure–time curve) represents the reaction rate. Embedding CNTs or CNT/Co_3_O_4_ onto the AP surface simultaneously enhances both peak pressure and pressurization rate. AP@CNT and AP@CNT/Co achieve peak pressures of 4.56 and 4.59 MPa, 5.8% and 6.5% higher than that of AP, respectively. Accordingly, their pressurization rates increase to 19.0 and 27.0 MPa s^−1^, 1.6-fold and 2.3-fold greater than that of AP, respectively. Higher pressurization rates realized by surface embedding over pristine AP confirm the promotion of thermal conduction pathways in energy release for AP composites. Moreover, given similar peak pressures, the superior pressurization rate of AP@CNT/Co over AP@CNT highlights the contribution of Co_3_O_4_ in catalyzing AP decomposition and accelerating gas generation.

Based on these findings, an energy release enhancement mechanism for AP@CNT/Co is proposed, centered on the surface thermal conduction pathways and porous architecture, as schematized in Fig. 8D. Pathway I relies on the thermal conduction pathways constructed on the AP surface. It is reported that a CNT possesses an axial thermal conductivity as ultrahigh as 3,000 W m^−1^ K^−1^ [46]. Embedding CNTs as a scaffold constructs efficient thermal conduction pathways on the AP surface, facilitating rapid heat flows from the surroundings, enhancing overall thermal conductivity and boosting reaction efficiency. Pathway II depends on the catalytic activation coming from Co_3_O_4_ nanoclusters anchored on CNTs. Pronounced catalytic activity arises from facilitated proton/electron transfer and oxidation, stemming from the unique crystal structure of Co_3_O_4_, mixed-valence state consisting of Co^3+^/Co^2+^, electronic configuration, and surface properties. Critically, there are 2 Co^3+^ units occupying octahedral sites in the Co_3_O_4_ crystal, resulting in strong oxidative capacity. Furthermore, the coexistence of mixed-valence states, surface-adsorbed oxygen species, and oxygen vacancies has been proven effective for lowering the energy barrier for electron transfer [47,48] and enabling high catalytic activity. Pathway III derives from the irregular porous structure formed via water droplet etching during processing and is essentially an enhanced mass transport. An increased specific surface area for AP@CNT/Co is realized, providing sufficient channels for heat/mass transfer and gaseous product diffusion, markedly accelerating reaction kinetics.

Overall, the strategy presented herein offers several advantages for achieving superior energy release, including synergistic multifunctionality that combines high thermal conductivity with efficient precision catalysis, effective preservation of the AP particle size to prevent agglomeration, and mitigation of hygroscopicity. The concerted action of these effects underpins the enhanced energy release of the AP@CNT/Co composite.

## Conclusion

In summary, this work presents an effective interface engineering strategy to address the inherent conflict between high catalytic activity and low additive loading for AP. The principal conclusions are as follows:

• Heterostructured CNT/Co_3_O_4_ was synthesized via in situ growth and embedded onto the AP surface via spray drying-suspension coating technology, preparing an AP@CNT/Co composite with a porous structure. A 15.4% enhancement in thermal conductivity and improved hygroscopicity were realized compared to those of pristine AP.
• The thermal decomposition of AP was significantly enhanced with a CNT/Co_3_O_4_ loading of 1 wt%, transitioning from a 2-stage exothermic process to a single, concentrated exothermic event. The temperature separation between LTD and HTD peaks was dramatically narrowed from 110.9 °C (pristine AP) to 12.7 °C. Meanwhile, a 64.8% reduction in decomposition activation energy and a 24.3% increase in heat release occurred, conclusively demonstrating that the precision catalysis strategy markedly outperforms conventional physical mixing.
• Energy release studies revealed more vigorous combustion and reduced agglomeration for AP@CNT/Co mixed with Al, attributable to accelerated reaction kinetics and enhanced oxidation of gaseous products. Compared to those of pristine AP, flame infrared radiation intensity increased by 72.6%, and the pressurization rate surged by 2.3 times.
• A synergistic enhancement mechanism for AP energy release is proposed: The concerted contribution of engineered thermal conduction pathways, precision catalysis, and the porous architecture optimizes heat/mass transportation and electron/proton transfer pathways within the AP@CNT/Co composite. This synergy enables rapid thermal conduction and high-efficiency catalysis, leading to substantially enhanced energy release for AP.

## Materials and Methods

### Starting materials

Multiwalled CNTs (99%), cobalt(II) nitrate hexahydrate (Co(NO_3_)_2_·6H_2_O, 99%), aqueous ammonia solution (NH_3_·H_2_O, 25 wt%), and potassium nitrate (KNO_3_, 99%) were supplied by Aladdin Reagent Co., Ltd. AP (industrial grade II) was obtained from Dalian North Potassium Chlorate Co., Ltd. Ethanol (AR) and deionized water were purchased from Sinopharm Chemical Reagent Co., Ltd.

### Synthesis of heterostructured CNT/Co_3_O_4_

Heterostructured CNT/Co_3_O_4_ was synthesized following a hydrothermal method [32]. Briefly, a measured quantity of Co(NO_3_)_2_·6H_2_O was dissolved in deionized water to prepare a 1 mol l^−1^ solution, which was transferred to a 10-ml volumetric flask and diluted to the mark with deionized water. Separately, 11 mg of CNTs was dispersed in 11 ml of deionized water via 30-min sonication to yield a 1 mg ml^−1^ suspension. The Co(NO_3_)_2_ solution was then mixed with NH_3_·H_2_O at a 1:2 volume ratio, followed by addition of the CNT suspension and 2.5 mol of KNO_3_. The mixture was magnetically stirred at room temperature for 4 h. Subsequently, the solution was transferred to a 25-ml Teflon-lined stainless-steel autoclave and hydrothermally treated at 180 °C for 12 h. After cooling to ambient temperature, the product was collected by centrifugation, washed repeatedly with deionized water and ethanol, and dried at 65 °C to obtain heterostructured CNT/Co_3_O_4_.

### Preparation of AP@CNT

AP@CNT composites were fabricated via spray drying-suspension coating technology, adapted from a reported protocol for AP@Al composites [45]. This technique exploits partial dissolution of AP surfaces under small droplet erosion, enabling CNT adhesion. Subsequent evaporation under hot airflow induces recrystallization of dissolved AP, encapsulating adhered CNTs. Specifically, a 0.01 g ml^−1^ CNT suspension was prepared in deionized water via 30-min sonication. Dried AP powder was fluidized in the chamber under a 95 °C hot airflow. The CNT suspension was injected into the chamber at 2 ml min^−1^ via a peristaltic pump. Resultant black solids (denoted AP@CNT) were collected after thorough drying. Samples extracted at equivalent time intervals during processing were designated I to V, with sample V (final product) containing 1 wt% CNTs.

### Preparation of AP@CNT/Co

The AP@CNT/Co composite was synthesized identically to AP@CNT, substituting CNTs with heterostructured CNT/Co_3_O_4_. Time-interval samples I to V were similarly obtained, with sample V containing 1 wt% CNT/Co_3_O_4_.

### Preparation of physical mixtures

For comparative studies, physical mixtures of AP with CNTs (AP+CNT) or CNT/Co_3_O_4_ (AP+CNT/Co) were prepared to address the effect of Co_3_O_4_ and precision catalysis strategy on AP decomposition. AP and the additive (1 wt%) were combined in an agate mortar and homogenized via manual grinding for 15 min.

### Characterization and test methods

XRD patterns were acquired using a Bruker D8 ADVANCE diffractometer (Germany). FT-IR spectra were recorded on a Shimadzu IRTracer-100 spectrometer (Japan). XPS analysis was performed on a Thermo Scientific K-Alpha spectrometer (USA). Surface morphology and elemental mapping were examined via SEM on a Zeiss Sigma 300 microscope (Germany) at an accelerating voltage of 10 kV. The internal structures of CNTs and heterostructured CNT/Co_3_O_4_ were characterized by TEM on a JEOL JEM-2100F instrument (Japan). The specific surface area was determined by nitrogen physisorption using a Micromeritics ASAP 2460 analyzer (USA). Prior to analysis, samples were degassed under vacuum at 65 °C for 12 h to remove adsorbed moisture and gases. Water contact angles were measured on a Lauda LSA 100 goniometer (USA) at 25 °C, using pressed sample pellets prepared at 1 MPa. A 5-μl deionized water droplet was gently deposited onto the surface of the pellets, and the evolution of the contact angle was recorded to assess hygroscopicity.

The thermal decomposition behavior of samples was evaluated by simultaneous DSC/TG on a Netzsch STA 449 instrument (Germany) under a 50 ml·min^−1^ argon flow, at a heating rate of 10 °C min^−1^. Evolved gaseous product analysis during decomposition was conducted using a Bruker Tensor II FT-IR spectrometer (Germany). The relative contents of the various gaseous products were determined by the peak absorbance intensity of their characteristic absorption bands in the FT-IR spectra. The thermal conductivity of compressed sample pellets (12.7-mm diameter, Fig. S9) was measured at 25 °C under a 20 ml min^−1^ argon flow using a Netzsch LFA 467 HyperFlash laser flash apparatus (Germany). The pressurization rate of the samples was recorded by a bomb calorimeter (ZDHW-HN7000C, Hebi Huaneng Electronic Technology Co., LTD, China), under an initial argon atmosphere of 3 MPa at room temperature (25 °C).

Combustion performance was assessed via a self-built multifunctional combustion diagnostic system (Fig. S10, detailed schematics in the Supplementary Materials), acquiring flame structure and radiation intensity data. The sample (AP, AP@CNT, or AP@CNT/Co) was thoroughly mixed with Al powder in a 1:1 mass ratio using a mortar and pestle for 15 min to ensure homogeneity. Approximately 1.0 g of the mixture was loosely loaded into a quartz crucible. The combustion test was carried out under an initial argon atmosphere of 1 MPa at room temperature (25 °C). Flame propagation and structure were recorded by a high-speed camera. Infrared radiation intensity was monitored using a high-speed infrared camera.

## Data Availability

All data needed to evaluate the conclusions of the study are present in the paper and Supplementary Materials.

## Supplementary Materials

Supplementary 1 Figs. S1 to S10 Table S1 Videos S1 and S2
