# Supplementary material for: Superior Energy Release of Ammonium Perchlorate Composites by Embedding Heterostructured Carbon Nanotube/Tricobalt Tetraoxide Thermal Conduction Pathways
Source: Research (Wash D C). 2025 Oct 15;8:0938. doi: 10.34133/research.0938 (PMC13248698; doi:10.34133/research.0938)
Supplement: Supplementary 1 — Figs. S1 to S10 Table S1 Videos S1 and S2 [file research.0938.f1.zip › Supplementary-materials.docx]

# *Supplementary Materials*

***for***

**Superior Energy Release of Ammonium Perchlorate Composites by Embedding Heterostructured Carbon Nanotube/Tricobalt Tetraoxide Thermal Conduction Pathways**

Ruixuan Xu^1,2^, Yuan Qin^3^, Junlian Hao^3^, Yongqiang Guo^1^, Hao Jiang^1^, Kejuan Meng^2^, Sulan Yang^4^, Kaili Zhang^2*^, Junwei Gu^1*^

*^1^ School of Chemistry and Chemical Engineering, Northwestern Polytechnical University, Xi'an, Shaanxi, 710072, P. R. China*

*^2^ Department of Mechanical Engineering, City University of Hong Kong, Hong Kong, 999077, P. R. China*

*^3^ National Key Laboratory of Solid Rocket Propulsion, Northwestern Polytechnical University, Xi'an, Shaanxi, 710072, P. R. China*

*^4^ Aerospace College, University of Electronic Science and Technology of China, Chengdu, Sichuan, 611731, P. R. China*

*Address correspondence to: [kaizhang@cityu.edu.hk](mailto:kaizhang@cityu.edu.hk) (K.Z.); [gjw@nwpu.edu.cn](mailto:gjw@nwpu.edu.cn) & [nwpugjw@163.com](mailto:nwpugjw@163.com) (J.G.)

# S1 Morphologies of samples


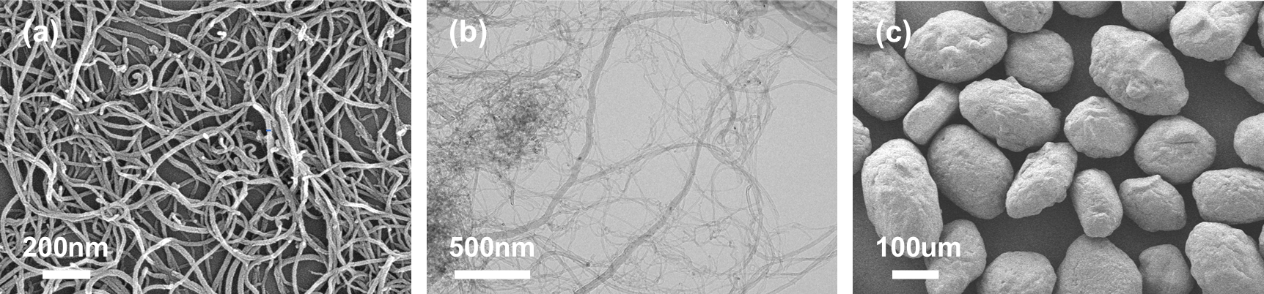


**Fig. S1** (a) SEM and (b) TEM images of CNT; (c) SEM image of AP.


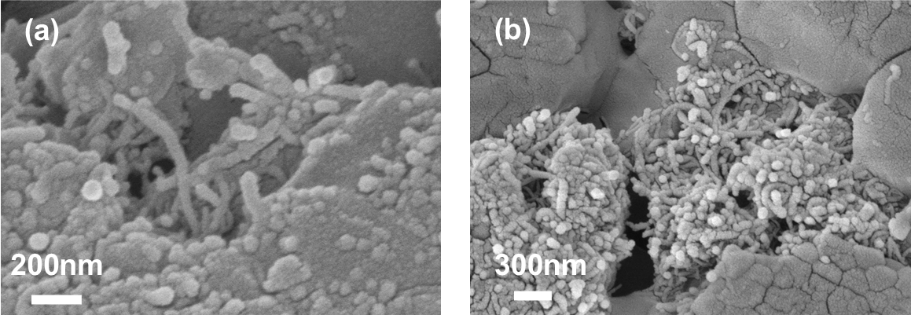


**Fig. S2** Magnified surface images of the composites: (a) AP@CNT/Co and (b) AP@CNT.


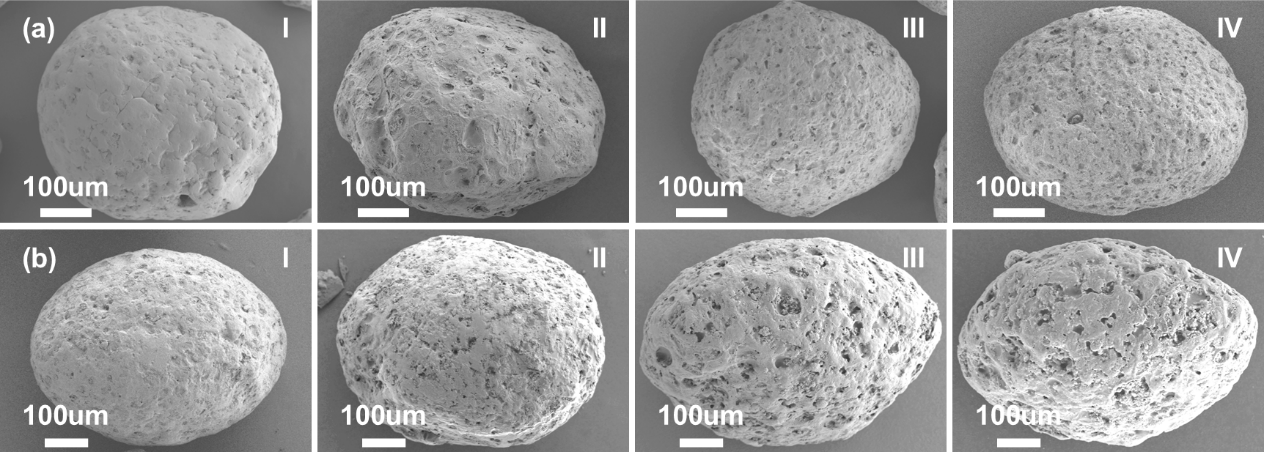


**Fig. S3** SEM images of composites sampled at equivalent time intervals: (a) AP@CNT/Co and (b) AP@CNT.

# S2 Water contact angle testing


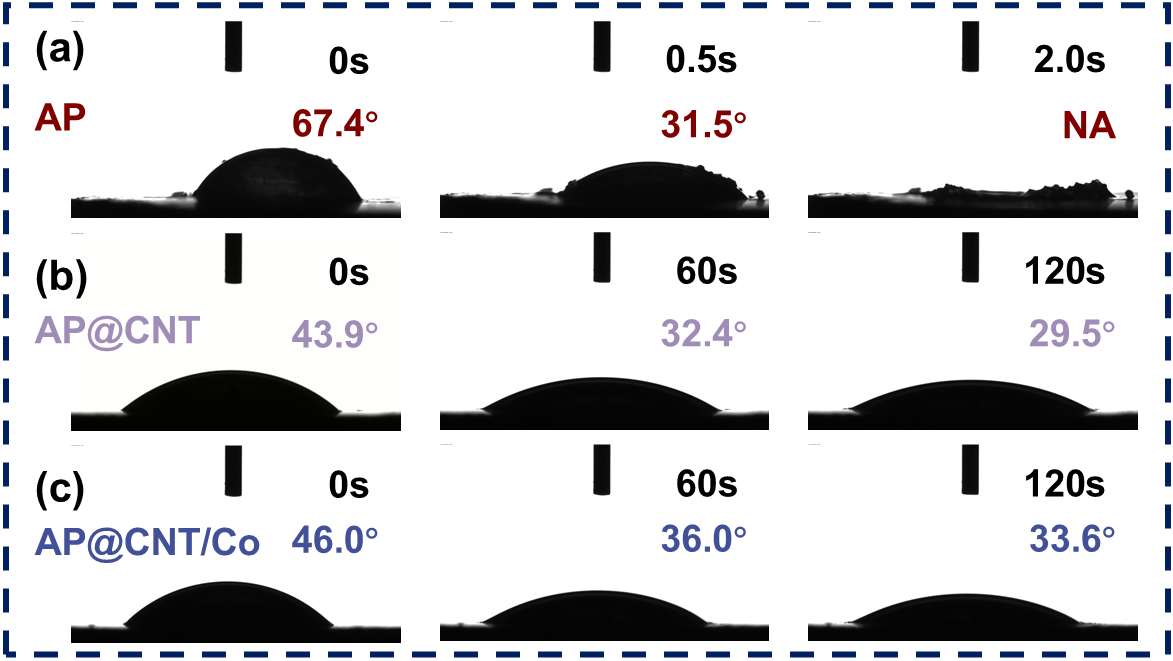


**Fig. S4** Water contact angle measurements: (a) AP, (b) AP@CNT, and (c) AP@CNT/Co.

# S3 Thermal decomposition behaviors


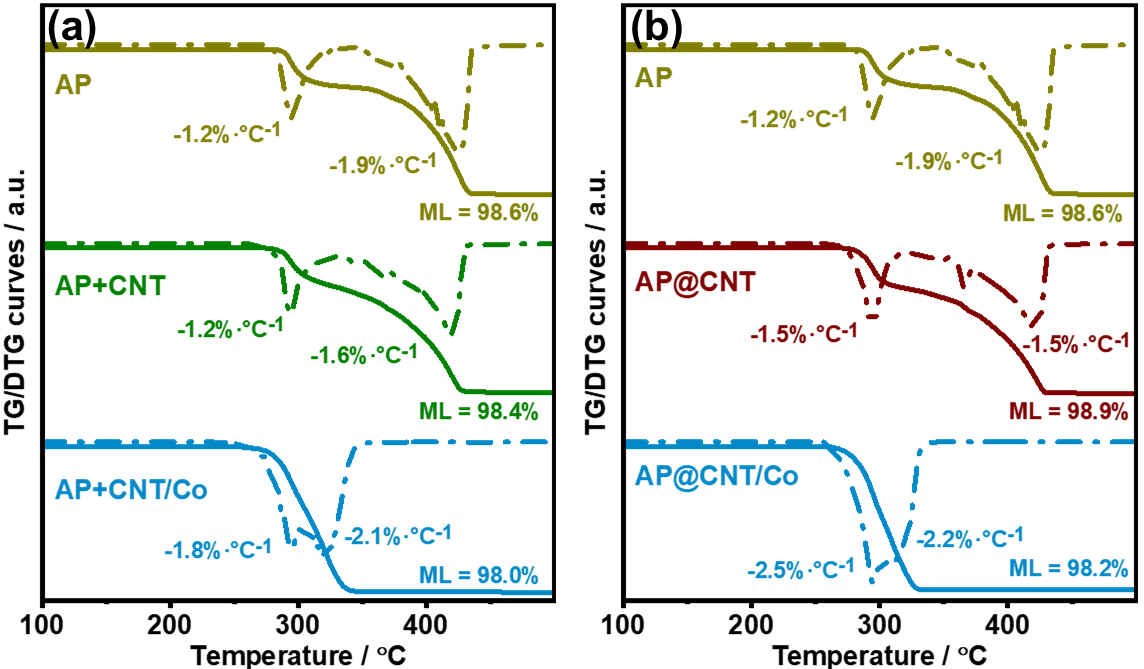


**Fig. S5** TG-DTG profiles of as-prepared samples: (a) AP, AP+CNT, and AP+CNT/Co; (b) AP, AP@CNT, and AP@CNT/Co.

**Table S1** Summary of kinetics parameters for the decomposition of samples.

| **Samples** | **Stage** | ***m*** | ***n*** | ***E*_a1_** | ***E*_a2_** | ***E*_a3_** |
| --- | --- | --- | --- | --- | --- | --- |
| AP | LTD | 0.497 | 0.887 | 108.5 | 113.1 | 116.3 |
|  | HTD | 0.614 | 0.712 | 222.3 | 235.8 | 240.9 |
| AP@CNT | LTD | 0.584 | 0.702 | 98.6 | 102.1 | 108.2 |
|  | HTD | 0.662 | 0.695 | 156.9 | 155.4 | 168.5 |
| AP@CNT/Co | - | 0.853 | 0.629 | 109.2 | 110.5 | 125.6 |

Note: LTD and HTD represent the low-temperature and high-temperature decomposition stage, respectively; *E*_a1_, *E*_a2_ and *E*_a3_ refer to the activation energy calculated by combined kinetic method, Friedman method (average value within 0.2＜*α*＜0.8, *α* means the conversion rate) and Kissinger method respectively, in kJ mol^-1^; *m* and *n* represent the equation parameters for the combined kinetic method.

# S4 Gaseous products


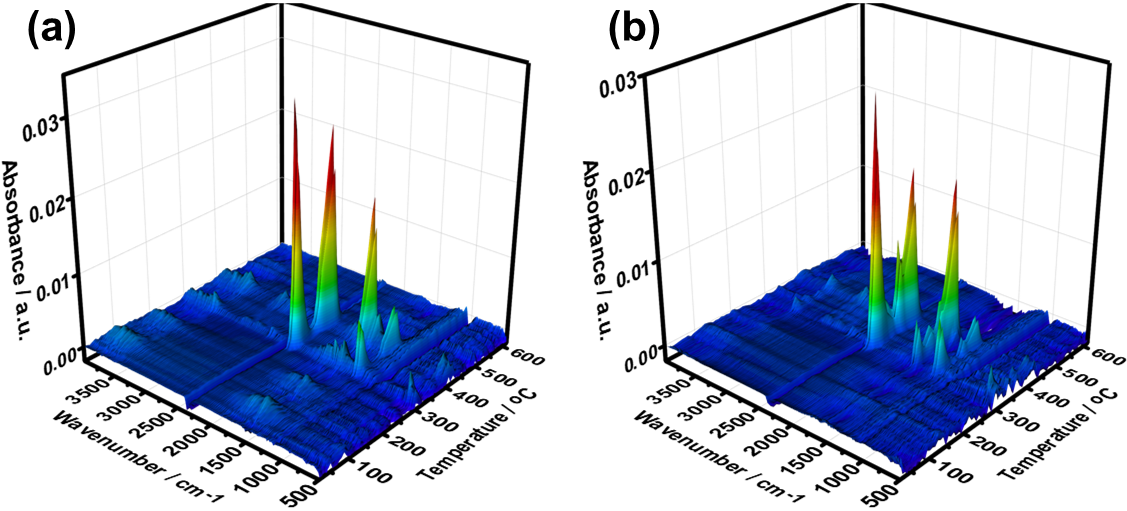


Fig. S6: Full-range spectra of gaseous products: (a) AP and (b) AP@CNT.


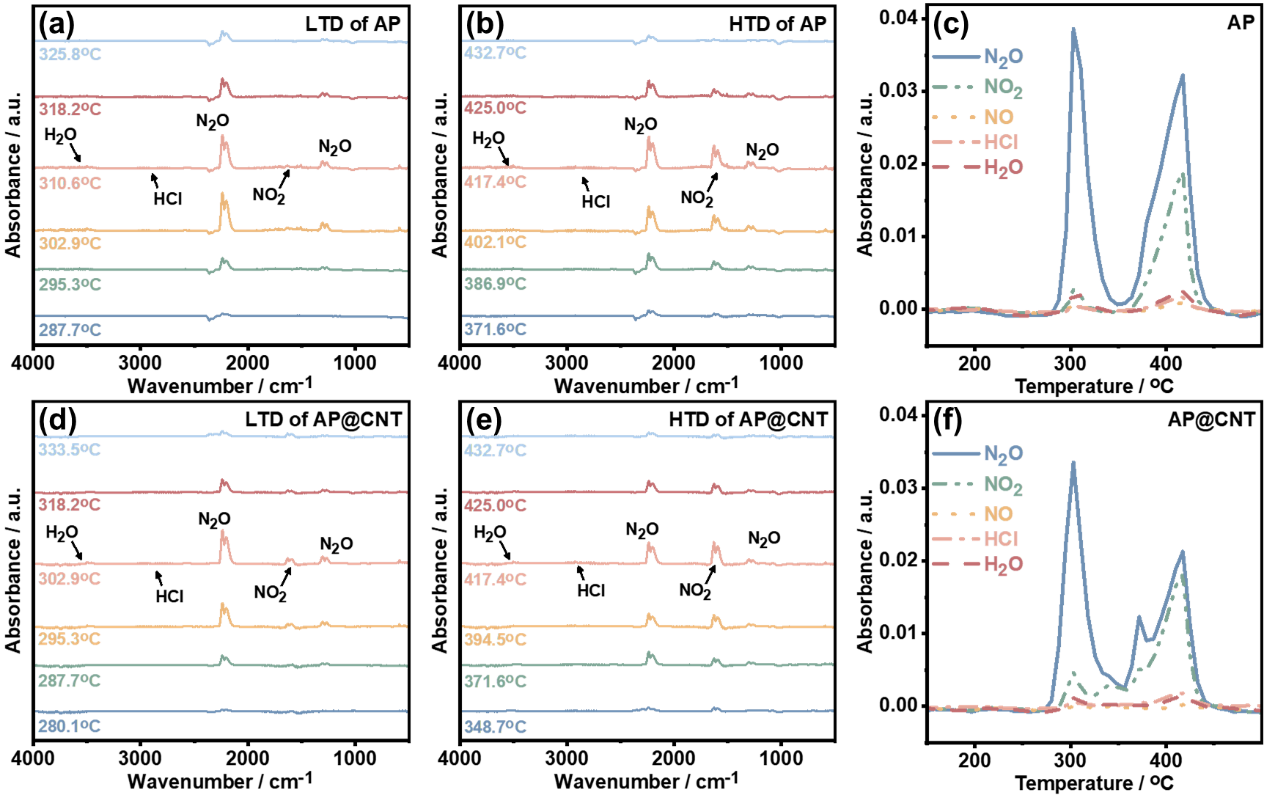


**Fig. S7** FT-IR analysis of gaseous products: (a, b) spectra of AP during LTD and HTD stages; (d, e) spectra of AP@CNT during LTD and HTD stages; (c, f) evolution profiles of three typical nitrogen oxides for AP and AP@CNT.

# S5 Flame structure and radiation intensity


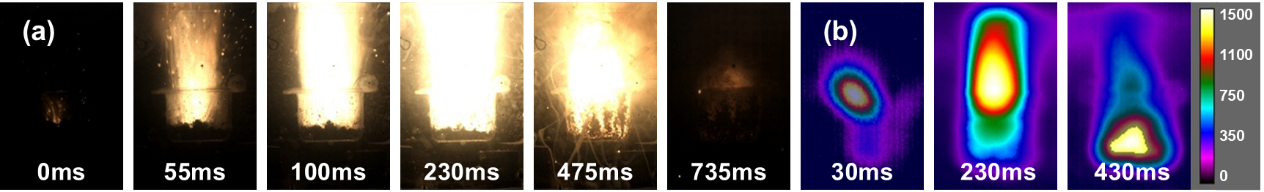


**Fig. S8** (a) Flame sequences and (b) infrared radiation images of AP@CNT composites mixed with equal-mass Al powders.

# S6 Pelletized samples for thermal conductivity testing


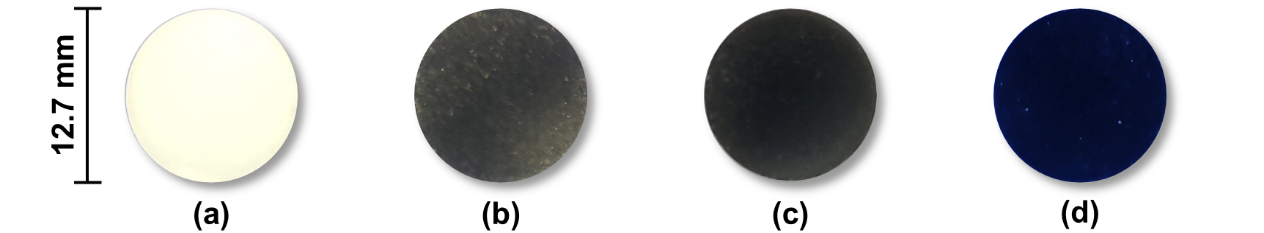


**Fig. S9** Photos of tablets for thermal conductivity tests: (a) AP, (b) AP+CNT, (c) AP@CNT and (d) AP@CNT/Co.

# S7 Combustion Diagnostic System Specifications

The multifunctional combustion diagnostic system comprises: (1) A constant-volume combustion chamber with sapphire viewports, (2) ignition electrodes connected to a NiCr wire, (3) high-pressure argon supply with pressure gauge, (4) high-speed/infrared cameras. The chamber maintains a sealed argon atmosphere at preset initial pressures. Flame propagation was captured through the sapphire viewports using high-speed/infrared cameras. Post-test pressure release was achieved via a dedicated exhaust port.


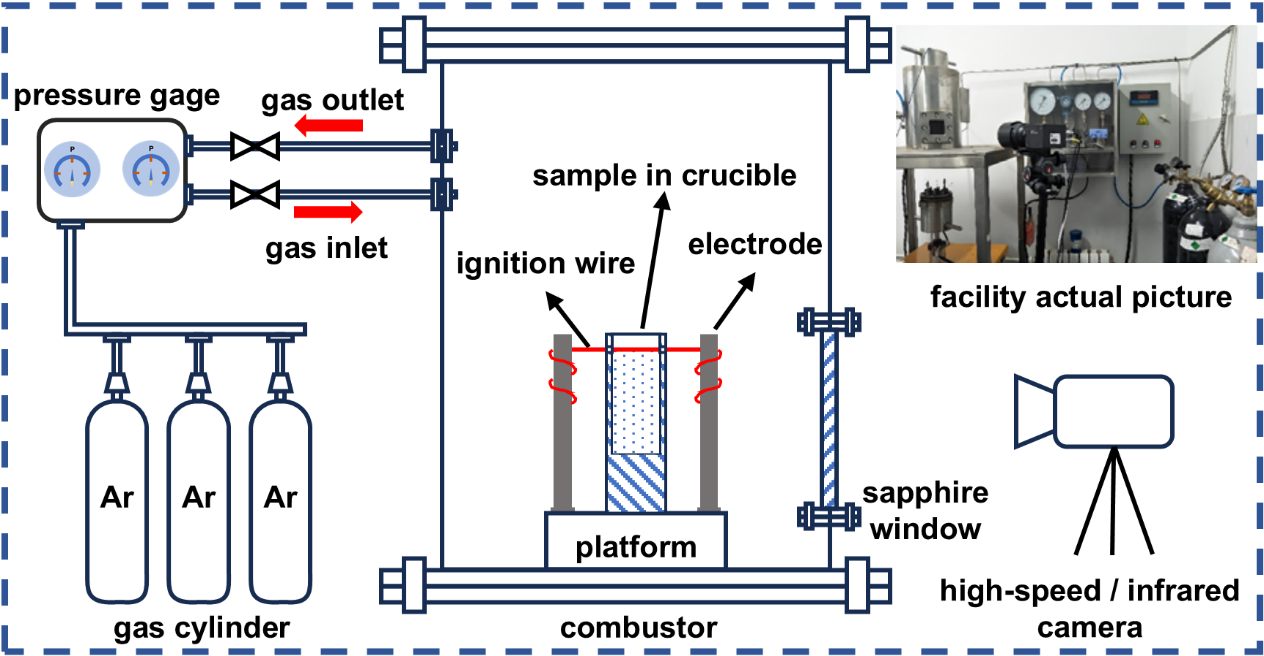


**Fig. S10** Schematic diagram of the composition for the multi-functional combustion diagnosis system.
