# Supplementary figures and images for: Superior Energy Release of Ammonium Perchlorate Composites by Embedding Heterostructured Carbon Nanotube/Tricobalt Tetraoxide Thermal Conduction Pathways
Source: Research (Wash D C). 2025 Oct 15;8:0938. doi: 10.34133/research.0938 (PMC13248698; doi:10.34133/research.0938)

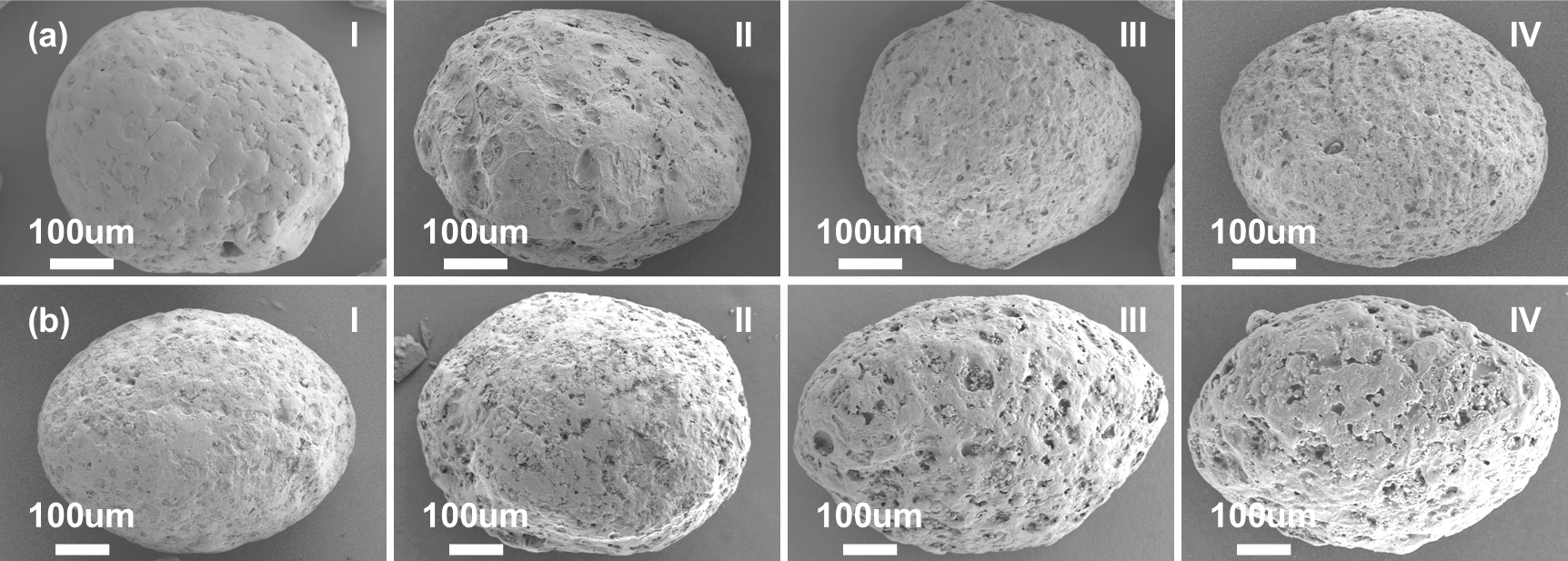

Supplement: Supplementary 1 — Figs. S1 to S10 Table S1 Videos S1 and S2 [file research.0938.f1.zip › FigureS3.TIF]

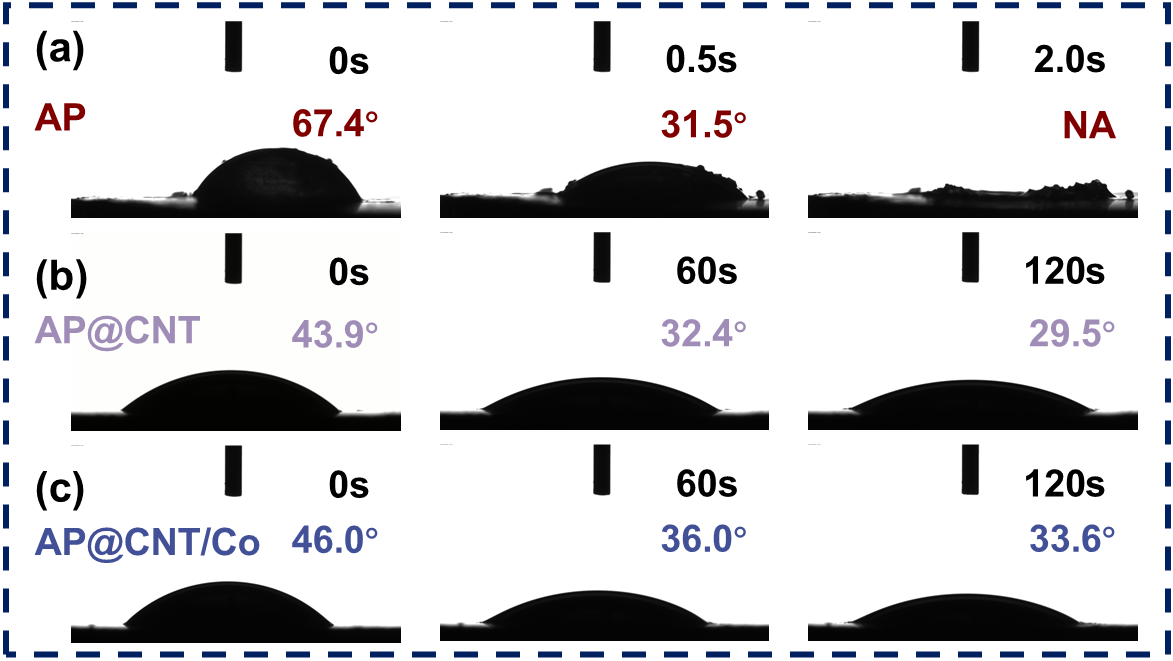

Supplement: Supplementary 1 — Figs. S1 to S10 Table S1 Videos S1 and S2 [file research.0938.f1.zip › FigureS4.TIF]

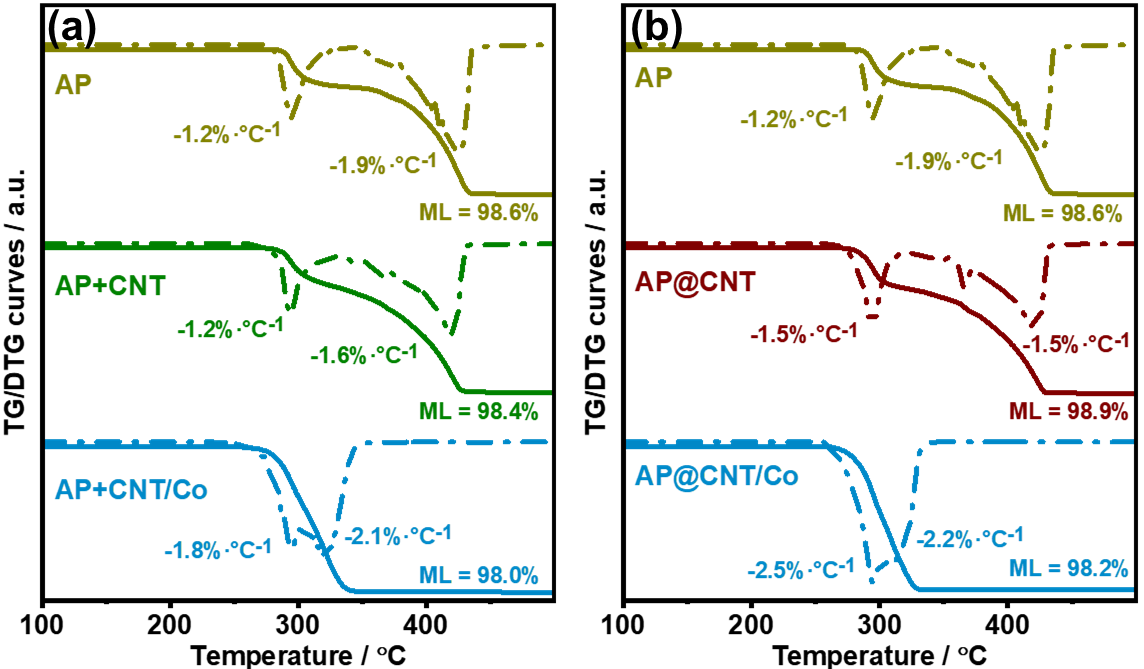

Supplement: Supplementary 1 — Figs. S1 to S10 Table S1 Videos S1 and S2 [file research.0938.f1.zip › FigureS5.TIF]

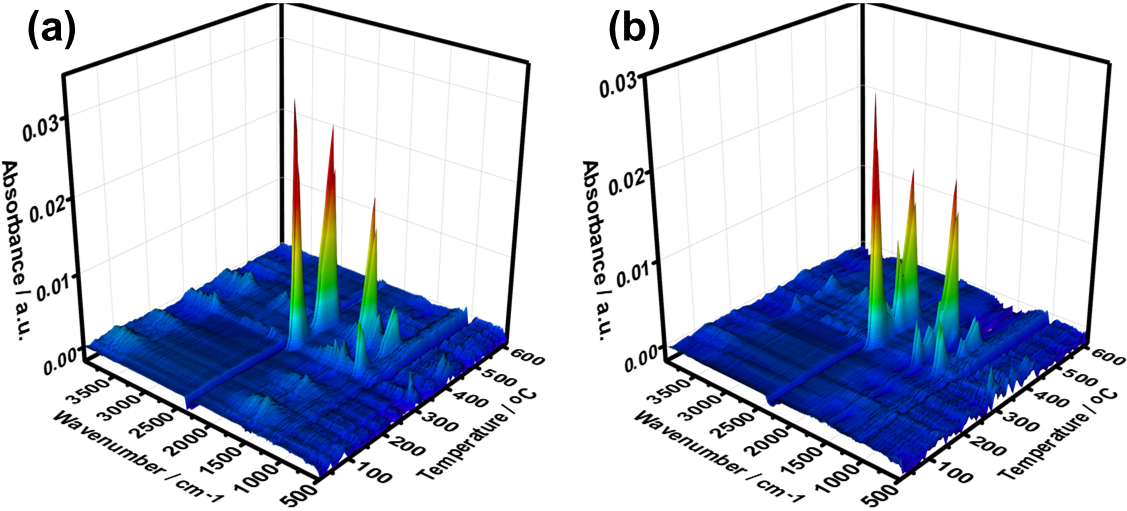

Supplement: Supplementary 1 — Figs. S1 to S10 Table S1 Videos S1 and S2 [file research.0938.f1.zip › FigureS6.TIF]

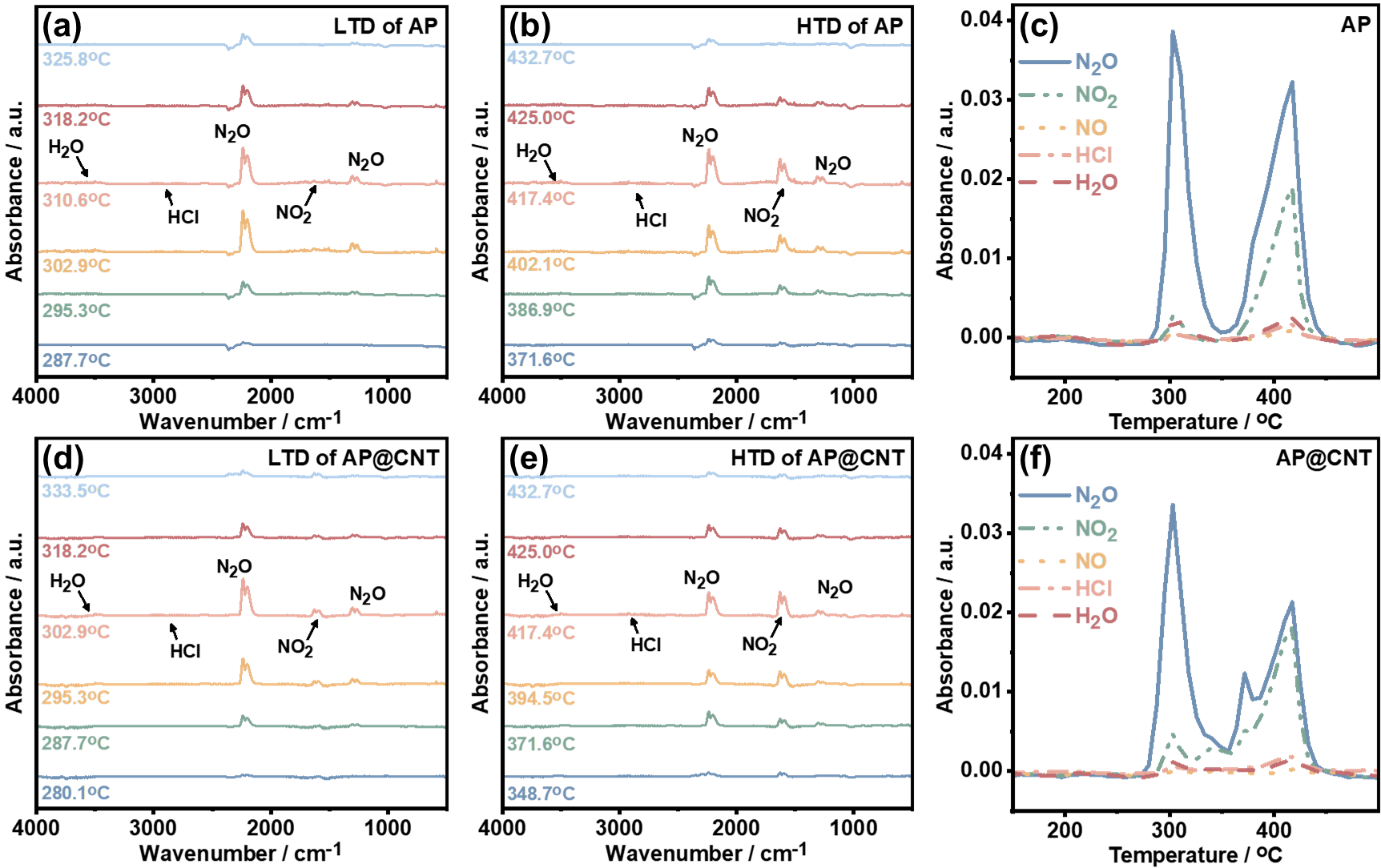

Supplement: Supplementary 1 — Figs. S1 to S10 Table S1 Videos S1 and S2 [file research.0938.f1.zip › FigureS7.TIF]

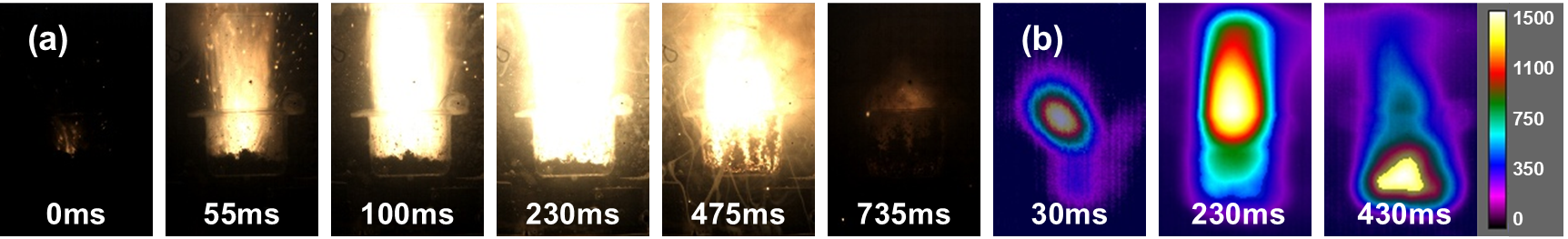

Supplement: Supplementary 1 — Figs. S1 to S10 Table S1 Videos S1 and S2 [file research.0938.f1.zip › FigureS8.TIF]

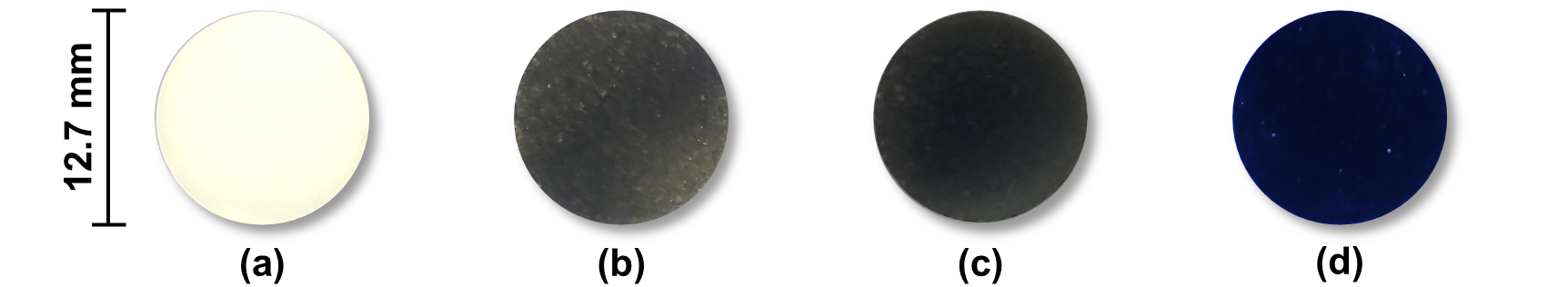

Supplement: Supplementary 1 — Figs. S1 to S10 Table S1 Videos S1 and S2 [file research.0938.f1.zip › FigureS9.TIF]

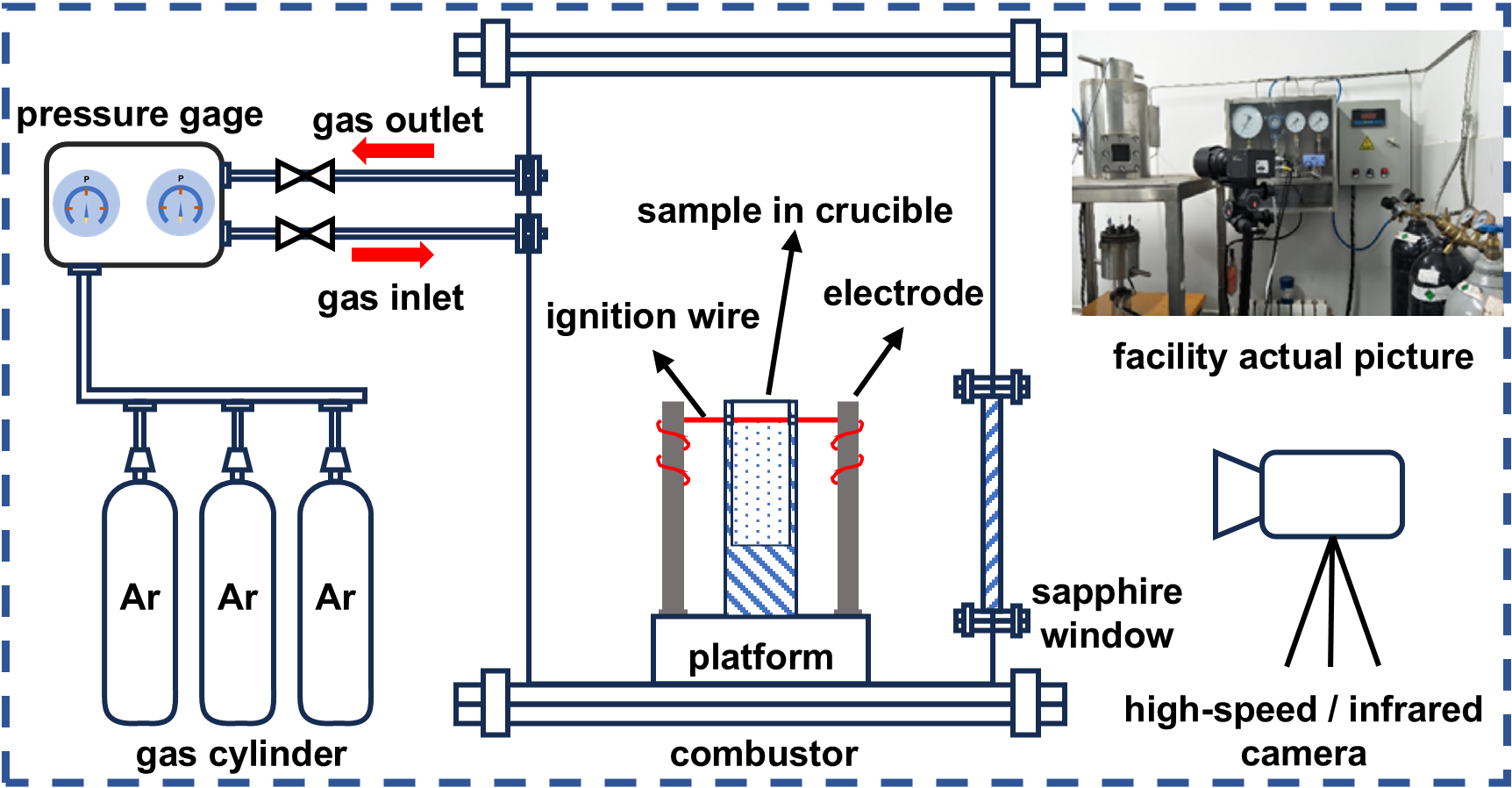

Supplement: Supplementary 1 — Figs. S1 to S10 Table S1 Videos S1 and S2 [file research.0938.f1.zip › FigureS10.tif]

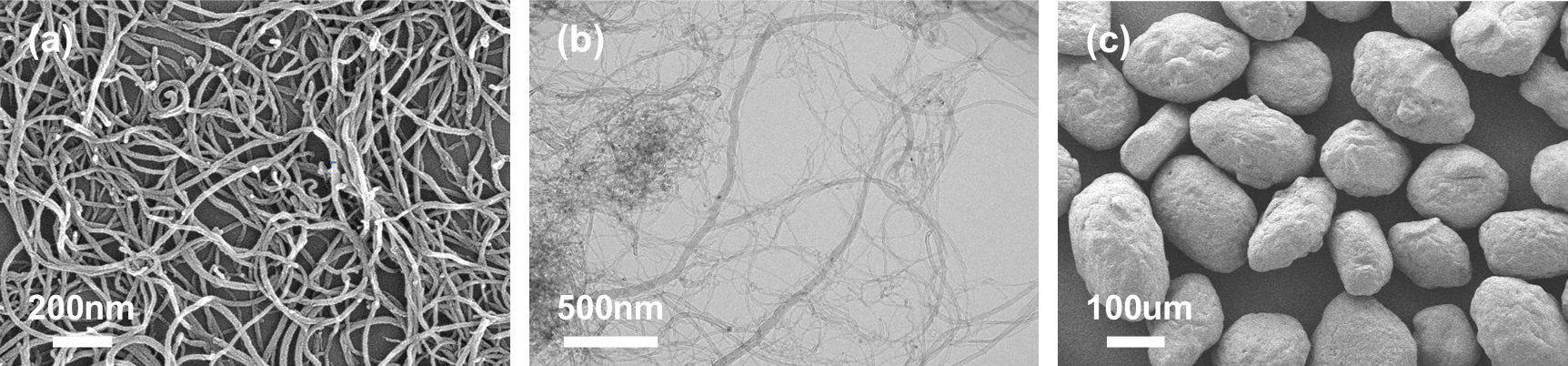

Supplement: Supplementary 1 — Figs. S1 to S10 Table S1 Videos S1 and S2 [file research.0938.f1.zip › FigureS1.TIF]

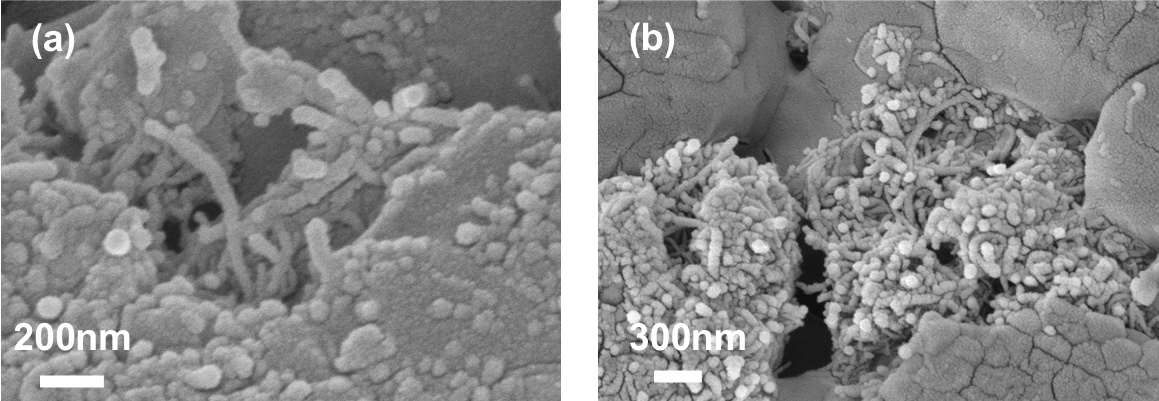

Supplement: Supplementary 1 — Figs. S1 to S10 Table S1 Videos S1 and S2 [file research.0938.f1.zip › FigureS2.TIF]
